# The impact of impulsivity and compulsivity on error processing in different motivational contexts

**DOI:** 10.3758/s13415-025-01281-5

**Published:** 2025-03-05

**Authors:** Rebecca Overmeyer, Tanja Endrass

**Affiliations:** 1 Faculty of Psychology, Institute of Clinical Psychology and Psychotherapy, Chair for Addiction Research, Technische Universität Dresden, Dresden, Germany; 2 Neuroimaging Center, Technische Universität Dresden, Dresden, Germany

**Keywords:** Impulsivity, Compulsivity, Performance monitoring, Motivational context, ERN

## Abstract

Neural correlates of performance monitoring, specifically the error-related negativity (ERN), are not only sensitive to motivation, but also altered in mental disorders marked by high levels of impulsivity and compulsivity. We explored the relationship between the ERN and individual differences in impulsivity and compulsivity. A total of 221 participants were recruited along the dimensions of impulsivity and compulsivity, and they performed a flanker task with a potential gain and a loss avoidance motivational context to assess error-related brain activity. We examined the ERN and theta power. Single trial regression was employed to analyze effects of motivational context and the relation to impulsivity and compulsivity. High impulsivity and compulsivity predicted higher ERN amplitudes within the gain context, but not the loss context. The interaction between both resulted in ERN amplitudes in the gain context being largest when impulsivity was high and compulsivity was low, and smallest when both were low. The ERN amplitude difference between gain and loss trials was highest if both impulsivity and compulsivity were low. Results indicate that both impulsivity and compulsivity are associated with larger ERN in the gain context, probably indicating higher subjective error significance. Both show a reduced modulation of the ERN with motivational context suggesting deficits in adaptive regulation of performance monitoring. Exploring transdiagnostic markers and their interactions could provide valuable insights into unraveling the complex dynamics that arise when examining the neural correlates of performance monitoring within the context of motivational effects.

**Supplementary Information:**

The online version contains supplementary material available at 10.3758/s13415-025-01281-5.

## Introduction

Adaptive behavior critically relies on performance monitoring to evaluate and effectively respond to errors: response outcomes are monitored for the need to adapt behavior to the current situation via the recruitment of cognitive control (Ullsperger et al., 2014a, 2014b; Ullsperger et al., 2014a, 2014b). Alterations in performance monitoring have been observed in mental disorders associated with impaired adaptive control, such as those marked by high impulsivity and compulsivity, e.g., obsessive compulsive disorder (OCD) and substance use disorder (SUD) (Endrass & Ullsperger, 2014; Euser et al., 2013; Gillan et al., 2016; Robbins et al., 2012). Given the transdiagnostic relevance of impulsivity and compulsivity across different disorders, we investigated their influence on neural correlates of performance monitoring in different motivational contexts.

A key component of performance monitoring is error monitoring, which involves the detection of errors to guide adaptive behavioral changes. A well-studied neural correlate of error monitoring is the error-related negativity (ERN), an event-related potential peaking at approximately 50 to 100 ms after erroneous responses at frontocentral locations (Falkenstein et al., 1991; Gehring et al., 1993; Ullsperger et al., 2014a, 2014b). The ERN, followed by an error positivity (Pe), is thought to reflect a fast alarm signal originating in the anterior midcingulate cortex (aMCC) indicating the need to adapt behavior (Debener et al., 2005; Steinhauser & Yeung, 2010; Ullsperger et al., 2010; Wessel et al., 2011). It has also been suggested to be influenced by subjective error significance (Endrass et al., 2010). The Pe is a later component associated with error awareness and the conscious evaluation of performance (Endrass et al., 2007; Ullsperger et al., 2014a, 2014b). The ERN may play an important role in the employment of cognitive control, in signaling for engagement of regulatory processes to adjust behavior (Kerns et al., 2004; Ridderinkhof et al., 2004). Critically, adaptive control is not only guided by internal and external goals, but via these goals also by motivation (Krebs & Woldorff, 2017). Moreover, the availability of rewards, as an external motivation, was shown to modulate complex cognitive functions, such as performance monitoring (for an overview, see Botvinick & Braver, 2015; Braver et al., 2014; Kouneiher et al., 2009), and consequently adjustments in cognitive control, guiding behavior, learning, and forward prediction (Desmurget & Grafton, 2000; Maurer et al., 2022; Vesper et al., 2017). Complementing this, the ERN was also suggested to be sensitive to motivation (Hajcak et al., 2005; Stürmer et al., 2011): more significant errors, as well as higher rewards, were reported to predict higher ERN amplitude. The ERN on punishment-motivated trials has been found to be elevated compared to gain-motivated trials. Loss aversion therefore appears to have an impact on performance monitoring (Potts, 2011). Alterations in ERN amplitude have been reported in mental disorders marked by impulsivity and compulsivity. Consistently, the ERN has been reported to be increased in individuals with OCD and high levels of compulsivity (Endrass et al., 2008, 2010; 2014; Endrass & Ullsperger, 2014; Mathews et al., 2012; Riesel et al., 2019b; Weinberg et al., 2015). Conversely, disorders characterized by high impulsivity, such as SUD and ADHD, as well as studies directly investigating impulsivity independent of psychopathology are mostly associated with attenuated ERN amplitude (Awasthi, 2022; Bellato et al., 2021; Checa et al., 2014; Franken et al., 2007; Luijten et al., 2014; Meyer & Hajcak, 2019; Riesel et al., 2019b; Smith & Mattick, 2013; Stahl & Gibbons, 2007; Takács et al., 2015; Taylor et al., 2018; Weinberg et al., 2015). However, increased ERN has been observed in individuals with alcohol use disorder that are abstinent or report comorbid anxiety disorders (Padilla et al., 2011; Schellekens et al., 2010).

In addition to the ERN, performance monitoring has been associated with increases in midfrontal theta activity, which is also linked to the aMCC (Debener et al., 2005; Keil et al., 2010). While both measures have been suggested to index performance monitoring (Yeung et al., 2004, 2007), they may reflect partially distinct and complementary aspects of the underlying neural processes (Beatty et al., 2020; Munneke et al., 2015). Theta power is thought to provide a broader view of oscillatory dynamics, which may reflect additional processes, such as cognitive control engagement (Eisma et al., 2021; Senoussi et al., 2022). Studies that include theta power as an additional measure therefore are able to provide a more comprehensive understanding of performance monitoring processes, assessing dynamic processes beyond those indexed by the ERN.

Consistently, alterations in neural correlates of performance monitoring have been reported in studies on disorders marked by impulsivity and compulsivity (Endrass & Ullsperger, 2014; Euser et al., 2013; Gillan et al., 2016; Robbins et al., 2012). Impulsivity and compulsivity are distinct but overlapping multidimensional constructs that are increasingly studied as dimensional phenotypes rather than categorical diagnostic markers (Chamberlain et al., 2018, 2019; Dalley et al., 2011; Fontenelle et al., 2022; Luigjes et al., 2019; Tiego et al., 2019). Impulsivity is characterized by rash and poorly planned behaviors and increased shifting (Robbins et al., 2012). Compulsivity is associated with rigid and persistent behaviors that overemphasize stability (Gillan et al., 2016; Robbins et al., 2012). Both constructs are associated with alterations in reward processing and motivation, inhibitory deficits, as well as clinical impairments (Dalley et al., 2011; Robbins et al., 2012). Examples of disorders commonly associated with impulsive, compulsive, or overlapping impulsive and compulsive symptoms are SUD and pathological gambling (Lee et al., 2019; Leeman & Potenza, 2012; Tiego et al., 2019), OCD (Frydman et al., 2020; Grassi et al., 2020; Kashyap et al., 2012; Parkes et al., 2019; Prochazkova et al., 2018), and eating disorders (Schaumberg et al., 2020; Solly et al., 2021; Waltmann et al., 2021). While impulsivity and compulsivity have been studied independently, growing evidence suggests their interaction may play an important role in shaping cognitive and behavioral outcomes, especially in the context of motivational influences, such as reward and punishment (Dalgleish et al., 2020; Ferreira et al., 2021; Tiego et al., 2019). Shared variance between these constructs has been identified as a predictor of co-occurring symptoms, underscoring the importance of transdiagnostic approaches to understanding their neural underpinnings (Tiego et al., 2019). Crucially, both impulsivity and compulsivity and associated symptoms are represented to varying degrees in the general population and have been associated with worse quality of life in young adults, even in the absence of a mental disorder (Chamberlain & Grant, 2019).

Because reward processing is altered in mental disorders marked by impulsivity and compulsivity (Figee et al., 2016; Martin & Potts, 2004; Novak et al., 2016), investigating possible motivational effects on error processing is crucial. Attenuated effects of error significance on ERN have been reported in individuals with OCD, with their error monitoring not differing between neutral and punishment conditions and a focus on cautious and error-avoidant response styles (Endrass et al., 2010; Riesel, Kathmann, et al., 2019a, 2019b). However, they are still sensitive to monetary incentives, emphasizing speed, and shifting the speed-accuracy tradeoff away from the pathological internal monitoring in tasks with monetary incentives (Banca et al., 2015; Riesel, Kathmann, et al., 2019a, 2019b). Altered sensitivity to anticipated rewards and losses in a medial prefrontal region has also been observed in OCD (Kaufmann et al., 2013). Reward-related attentional capture has been associated with severity of compulsivity transdiagnostically, and therefore across addictive and OCD-related behaviors, with higher compulsivity being associated with more attention toward signals of high reward (Albertella et al., 2019). Similar to the results on compulsivity, there has been a broad interest in the effect of impulsivity on performance monitoring. Individuals with low impulsivity have been found to exhibit higher ERN amplitudes on punishment-motivated trials, compared to gain-motivated trials. Individuals scoring high on impulsivity exhibited equivalent ERN amplitude across motivation type, but they exhibited attenuated ERN on punishment-motivated trials compared to low impulsive individuals. Threat of punishment therefore increased performance monitoring only in low impulsive individuals (Potts et al., 2006). Interestingly, individuals scoring low on a scale assessing socialization, a trait linked to impulsivity, showed smaller ERNs in punishment than reward contexts, indicating reduced error salience in aversive situations (Dikman & Allen, 2000). Similarly, based on the finding that the ERN was larger after high-risk choices in low but not high impulsive individuals, Martin and Potts (2009) conclude that highly impulsive individuals are less sensitive to the negative consequences of their choices. Motivational effects on the ERN have also been examined in smokers, who showed larger ERN in reward-motivated trials (Schlienz et al., 2013), but not for punishment-motivated trials (Potts et al., 2014). Few studies have investigated reward and punishment motivation jointly. Prior research therefore suggests that high compulsivity is associated with higher ERN amplitudes and less adaptation across motivational contexts, reflecting rigid response styles and heightened internal monitoring. Conversely, high impulsivity is associated with attenuated ERN amplitudes and higher impact of contexts with potential gain, potentially being less sensitive to punishment. Impulsivity and compulsivity may also interact in predicting the ERN amplitude (Hill et al., 2016).

The interaction between impulsivity and compulsivity may significantly influence performance monitoring, reflecting the interplay of rigid and disinhibited response styles in the face of motivational incentives. This study was designed to investigate the relationship between impulsivity, compulsivity, and neural markers of performance monitoring (ERN and theta power) across different motivational contexts. Performance monitoring was assessed within a non-clinical sample of adults using electroencephalography (EEG) in an adapted version of the arrow Flanker task (Eriksen & Eriksen, 1974; Kopp et al., 1996) with extrinsic motivational contexts. We expected higher ERN amplitudes and theta power at higher levels of compulsivity (positive association), reflecting increased error sensitivity. Conversely, we expected smaller ERN amplitudes and theta power at higher levels of impulsivity (negative association). Additionally, we hypothesized that the influence of motivational context would be more pronounced for individuals with high impulsivity compared to those with high compulsivity. Finally, we explored potential interactions between impulsivity and compulsivity and their impact on error-related brain activity.

## Methods

### Sample

We recruited 253 participants along the dimensions of impulsivity and compulsivity, as assessed by the Obsessive–Compulsive Inventory-Revised (OCI-R; Foa et al., 2002; Gönner et al., 2007) and Barratt Impulsiveness Scale (BIS-11; Patton et al., 1995), in the Dresden area. Thirty-two participants were excluded from further analyses: 12 participants made more than 40% errors across all trials, seven had a significant number of random button presses, 11 had less than 16 errors per motivational context (Clayson, 2020), one had bad data quality, and another had discontinued the assessment. See Supplement 1 for a more detailed description of recruitment procedures. The final sample consisted of 221 participants (46.6% female; M = 25.16 years, SD = 4.94); 207 participants (93.7%) had completed advanced education degrees.

A total of 25 participants (11.3%) reported a history of mental health problems. The most prevalent diagnoses were depression (n = 16) and anxiety disorders (n = 12), other diagnoses included posttraumatic stress disorder (n = 2), eating disorders (n = 2), OCD (n = 1), and somatoform disorders (n = 1). Comorbidity was present in nine participants (4.1%); 91.8% of participants self-reported as of mainly European, 5.9% as of mainly Asian or Middle Eastern ancestry, and 2.3% of unknown ancestry. See Table 1 for descriptive statistics of sample characteristics.

**Table 1:** Sample characteristics

	*M*	*SD*	*Range*
Impulsivity (BIS-11)	60.57	8.95	38–96
Compulsivity (OCI-R)	12.74	9.33	0–46
*Other impulsivity subfacets*			
UPPS urgency	26.31	5.86	14–43
UPPS lack of premeditation	22.38	4.36	13–38
UPPS lack of perseverance	19.38	4.29	10–31
UPPS sensation seeking	33.05	7.04	14–46
*Anxiety*			
Anxiety (DASS-21)	1.89	2.57	0–20
Somatic anxiety (STICSA)	14.40	2.84	11–26
Cognitive anxiety (STICSA)	15.81	3.98	10–32

BIS-11 = Barratt Impulsiveness Scale; OCI-R = Obsessive–Compulsive Inventory-Revised; UPPS = UPPS Impulsive Behavior Scale; DASS-21 = Depression Anxiety Stress Scales-21; STICSA = State-Trait Inventory of Cognitive and Somatic Anxiety

All participants had normal or corrected-to-normal vision, were native speakers of German, and reported no history of head trauma or neurological disease. Participants were not included if they: reported taking psychotropic substances within the past 3 months; reported a history of bipolar disorder, borderline personality disorder, psychotic episodes, or severe alcohol use disorder; currently met the criteria for an eating disorder or severe episode of major depression; reported a lifetime use of illicit substances of more than twice a year and lifetime use of cannabis of more than twice a month.

A sample of 223 was chosen to detect R^2^ changes in linear multiple regression models with three tested predictors with small effect sizes of f^2^ = 0.05 at α = 0.05 and a power of 80%. Results on a subsample have been published before (Overmeyer et al., 2021). The study was conducted in accordance with the ethical guidelines of the Declaration of Helsinki. The ethics committee at the University Hospital Carl Gustav Carus, Technische Universität Dresden approved study procedures (EK 372092017). All participants gave informed consent. Although we did not provide a formal preregistration of the project, the initial project proposal is available on OSF (https://osf.io/ywnze/).

### Procedure and measures

#### Procedure

Scores for the OCI-R and BIS-11 were assessed online, before participants completed two sessions in the lab, and between those sessions a week of ecological momentary assessment of self-control in daily life. Questionnaire data were obtained during the first session. Performance monitoring-related brain activity was assessed using the ERN during a monetary incentive version of the arrow flanker task (Eriksen & Eriksen, 1974; Kopp et al., 1996) using EEG during the second session. The EEG session took place at least 8 days after the first session owing to the duration of the ecological momentary assessment. During both sessions, participants completed other tasks, which are not part of this report.

#### Obsessive–compulsive inventory-revised

Compulsivity was measured using the OCI-R (Foa et al., 2002; Gönner et al., 2007). The OCI-R is a self-report measure, with 18 items resulting in a sum score of good internal consistency (α = 0.85) in its German adaptation (Gönner et al., 2007), and assesses obsessive–compulsive symptom dimensions (washing, checking, doubting, ordering, obsessing, hoarding, and mental neutralizing).

#### Barratt impulsiveness scale

Impulsivity was measured using a German translation of the BIS-11 (Patton et al., 1995). The BIS-11 is a self-report measure with 30 items resulting in a sum score of good internal consistency (α = 0.83; Stanford et al., 2009).

#### Other questionnaires

Participants also completed the UPPS Impulsive Behavior Scale (Schmidt et al., 2008), the anxiety subscale of the Depression Anxiety Stress Scales (Henry & Crawford, 2005; Nilges & Essau, 2015) and both trait subscales of the State-Trait Inventory for Cognitive and Somatic Anxiety (STICSA; Overmeyer & Endrass, 2023; Ree et al., 2008).

#### Monetary incentive flanker task

**Fig. 1:**
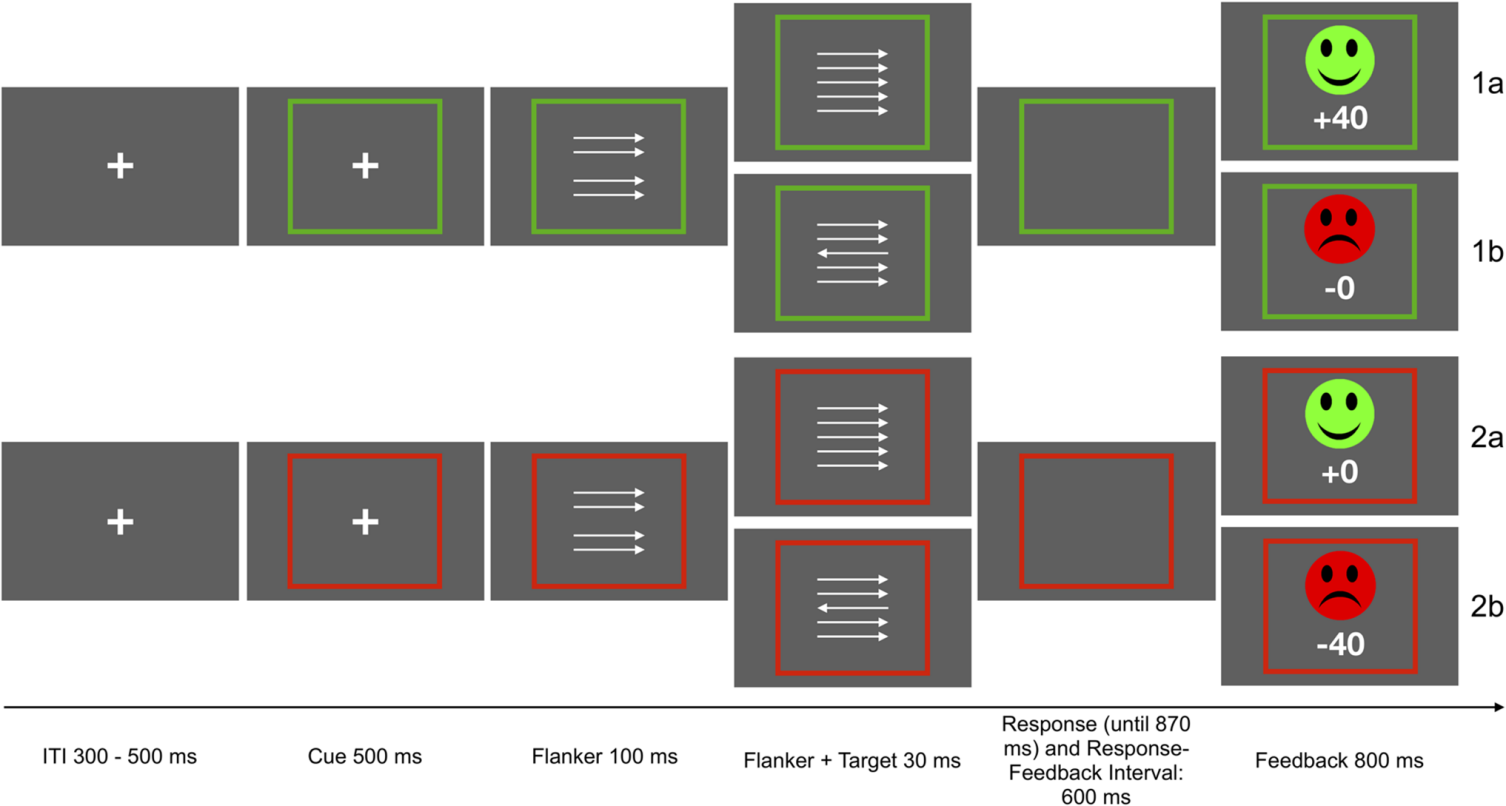
Schematic depiction of the Monetary Incentive Flanker Task. Participants were instructed to respond with the left or right button according to the direction of the target stimulus. The target stimulus was a middle arrow that appeared for 30 ms with a delay of 100 ms relative to the onset of the surrounding flanker arrows. In 50% of the trials, the target stimulus pointed in the same direction (congruent) as the surrounding arrows. In the other 50% of the trials, the target pointed in the opposite direction (incongruent). The incentive context was cued as follows: Each trial started with an incentive cue, a green or red frame surrounding a fixation cross, signaling potential gain (green) or loss (red) of 40 points in the current trial (presented for 500 ms). The frame remained visible throughout the trial. In the potential gain context (50% of all trials), the fastest 80% of the correct responses were rewarded (40 points, 1a) while errors and slowest responses resulted in reward omission (0 points, 1b). In the loss avoidance context, incorrect and the slowest responses were punished (minus 40 points, 2b), and the fastest 80% of correct responses resulted in punishment omission (0 points, 2a). Slow responses were defined by an adaptive deadline based on individual performance and response time, in order to obtain a rate of 20% negative feedback for each context. Performance feedback was presented for 800 ms after a response interval of 900 ms following target onset or 600 ms after response

Participants performed a monetary incentive flanker task, which is a modified version of the arrow-version of the Eriksen flanker task (Eriksen & Eriksen, 1974; Kopp et al., 1996). The flanker stimuli consisted of four vertically arranged arrows pointing to the left or the right. Participants had to respond using a left or right button, according to the direction of the target arrow (Fig. 1). Participants could earn a bonus of up to 5 EUR, depending on task performance and points earned. The task included 640 trials of 2.53 s to 2.75 s duration. The task was presented using Presentation 19.0 (Neurobehavioral Systems Inc., Berkeley, CA).

### Psychophysiological recording and data reduction

The EEG was continuously recorded at a sampling rate of 500 Hz using elastic EEG caps with 63 Ag/AgCl electrodes at equidistant locations (EasyCap GmbH, Herrsching-Breitbrunn, Germany) and two 32-channel BrainAmp amplifiers (Brain Products GmbH, Munich, Germany). Data were recorded by using high-pass and low-pass filters set at 0.1 to 250 Hz (Slope: 12db/Oct), while impedances were kept below 10 kOhm. Two external electrodes placed below the left and right eye were used to capture eye movement, in addition to electrodes at LO1 and LO2. Ground and reference electrodes were placed next to Fz (at AFF1h and AFF2h, theta/phi spherical coordinates: − 58/78 and 58/78). Offline analyses were performed using EEGLAB 14.1.2 (Delorme & Makeig, 2004) and MATLAB 2018b (The MathWorks Inc., 2018). The EEG was low and high pass filtered using a Hamming windowed sinc finite impulse response (FIR) filter with the lower edge of the pass band at 0.1 (cutoff frequency (− 6 dB): 0.05 Hz) and 30 (cutoff frequency (− 6 dB): 33.75 Hz) Hz (as recommended by Luck, 2014), respectively, and epoched from − 500 to 2000 ms relative to target stimulus onset. Epochs with artifacts were rejected automatically based on signal deviations greater than 5 SD of the mean probability distribution on any single channel or the whole montage (pop_jointprob). Behaviorally, trials with reaction times outside the range of 150—600 ms were rejected. Remaining epochs were demeaned and submitted to adaptive mixture independent component analysis (AMICA) implemented in EEGLAB. Independent components reflecting ocular or cardiovascular artifacts were removed manually, aided by the ICLabel toolbox (Pion-Tonachini et al., 2019), and EEG data were re-referenced to common average reference. Subsequently, response-locked epochs from − 500 to 1000 ms were created. The average EEG activity 200 to 0 ms prior to response was used as baseline. Individual participants’ mean amplitudes per trial type were calculated for each time point and electrode within the extracted epochs. Internal consistency of EEG measures was excellent as determined by the Spearman-Brown corrected split-half reliability (odd–even method) for error and correct amplitudes overall (0.96, 0.98), in the gain (0.93, 0.95) and the loss (0.91, 0.95) context for incongruent trials, respectively.

We also analyzed response-locked activity in the time–frequency domain for the theta band (3–7 Hz) and the delta band (1–3 Hz). Analyses were conducted according to recommendations by Cohen (2014). Data were epoched around the response onset (− 1000 to 2000 ms). The interval between − 500 to − 200 ms was used for baseline correction and automatic artifact rejection applied. EEG time series in each epoch was be convolved with a set of complex Morlet wavelets, defined as a Gaussian-windowed complex sine wave: ${e}^{-i2\pi ft}{e}^{\frac{-4\text{log}\left(2\right){t}^{2}}{{h}^{2}}}$, where t is time, f is frequency, which increases from 1 to 20 Hz in 20 logarithmically spaced steps, and h defines the full-width at half-maximum, which ranges from 600 to 300 ms with 20 logarithmically spaced steps (Cohen, 2019). Power was normalized by conversion to a decibel (dB) scale (10 ∗ log10[power(t)/power(baseline)]) to allow direct comparison of effects across frequency bands. Each epoch was then cut to − 200 to 600 ms.

### Data analysis

All analyses were performed by using MATLAB (The MathWorks Inc., 2018) and R (R Core Team, 2022), for used R packages see supplement 2.

#### Sample characteristics and behavioral analysis

To assess participants behavior in the task and to examine post-error slowing (PES), post-error increase in accuracy (PIA), and post-error speeding for incongruent trials (PERI; post-error reduction of interference) (Danielmeier & Ullsperger, 2011), we conducted two multiple robust single-trial regressions. We regressed critical task factors onto participant’s single-trial accuracy (GLM1) and reaction time (RT; GLM2) while controlling for confounds and the interdependence of effects (Fischer et al., 2018). Individual regression weights were compared using two-sided t-tests corrected for multiple comparisons on group level (see Supplement 3 for further details). Additionally, the effect of impulsivity, compulsivity and their interaction (regressors were scaled) on error rate, PIA, post-correct accuracy (PCA), PES and correct RT was tested using multiple linear regression analyses. Resulting *p* values were adjusted for multiple comparisons using the FDR procedure (Benjamini & Yekutieli, 2001).

#### ERP analysis

As the single-trial regression analyses examines effects for the error vs. correct contrast, we conducted an additional ERP analysis to specifically examine error-related ERPs. The ERN and CRN were determined as the mean activity within ± 10 ms of the individual peak (determined as the most negative peak within 0–150 ms after the response) in a cluster of frontocentral electrodes FCz and Cz. The early Pe was defined as the mean amplitude between 170 and 250 ms after response onset in a cluster of frontocentral electrodes FCz and Cz. The late Pe was defined as the mean amplitude between 300 and 500 ms after response onset in a cluster of parietal electrodes CPz and Pz. The effect of impulsivity, compulsivity and their interaction (regressors were scaled) on the event-related potentials was tested using multiple linear regression analyses (*ERN amplitude* = *b0* + *impulsivity x b1* + *compulsivity x b2* + impulsivity*compulsivity x b3 + *e)*. Resulting *p* values were adjusted for multiple comparisons (Benjamini & Yekutieli, 2001).

#### Single-trial analysis

Multiple robust regression (O’Leary, 1990) was employed to build a general linear model (GLM) and regress behavioral and task parameters on single-trial EEG activity at each electrode and time point (see Fischer & Ullsperger, 2013) using the following linear equation: *Y* = *b0* + *regressor1 x b1* + *regressor2 x b2* + *regressor3 x b3* + *…* + *e* (first level). The resulting individual b values were standardized by their SDs before averaging across subjects to penalize the regression model in case of multicollinearity of predictors and ensure comparability between predictors. We then conducted multiple linear regression analyses (second level) to compute the effects of impulsivity, compulsivity and their interaction (these continuous regressors were scaled) on standardized b values of a regressor from the first level for the response-locked model, for frontocentral and parietal electrodes (*regressor* = *b0* + *impulsivity x b1* + *compulsivity x b2* + impulsivity*compulsivity x b3 + *e*).

To examine how impulsivity, compulsivity, and their interaction influence the relationship between accuracy and motivational context in neural activity, we first built a model for response-locked EEG, which only included incongruent trials. The model included a regressor coding the accuracy of the current trial (correct/error), the context of the current trial (gain, loss) and their interaction; log scaled RT of the current trial and response hand (left/right) were additional regressors: *EEG*_*response*_ = *b0* + *accuracy x b1* + *context x b2* + accuracy*context x b3 + *logRT x b4* + *response hand x b5* + *e*. We then conducted multiple linear regression analyses to compute the effects of impulsivity, compulsivity and their interaction on standardized b values of the interaction regressor (accuracy*context), for frontocentral and parietal electrodes (accuracy*context = *b0* + *impulsivity x b1* + *compulsivity x b2* + impulsivity*compulsivity x b3 + *e*).

To examine how impulsivity, compulsivity, and their interaction influence the impact of motivational context on neural activity during error processing, we followed up with a model only including incongruent error trials (with regressors context, log scaled RT of the current trial, response hand: *EEG*_*error*_ = *b0* + *context x b1* + *logRT x b2* + *response hand x b3* + *e*) to analyze effects of context on the ERN. To separately examine how impulsivity, compulsivity, and their interaction influence the impact of accuracy on neural activity within each motivational context, two additional follow-up models with accuracy effects were analyzed separately by context: 1) a model including incongruent trials in the gain context (with regressors accuracy, log scaled RT of the current trial, response hand: *EEG*_*gain*_ = *b0* + *accuracy x b1* + *logRT x b2* + *response hand x b3* + *e*), and 2) a model including incongruent trials in the loss context (with regressors accuracy, log scaled RT of the current trial, response hand: *EEG*_*loss*_ = *b0* + *accuracy x b1* + *logRT x b2* + *response hand x b3* + *e*). We then conducted multiple linear regression analyses to compute the effects of impulsivity, compulsivity and their interaction on standardized b values of the *context* regressor for *EEG*_*error*_, for frontocentral and parietal electrodes (*context* = *b0* + *impulsivity x b1* + *compulsivity x b2* + impulsivity*compulsivity x b3 + *e*), as well as the standardized b values of the *accuracy* regressor for *EEG*_*gain*_ and *EEG*_*loss*_ for frontocentral and parietal electrodes (*accuracy* = *b0* + *impulsivity x b1* + *compulsivity x b2* + impulsivity*compulsivity x b3 + *e*). Additional feedback-locked analyses can be found in supplement 5. The significance threshold was corrected using a *p* value of 0.05/3.

#### Time–frequency analysis

The effect of impulsivity, compulsivity and their interaction (regressors were scaled) on time–frequency delta and theta of response-locked activity was also tested using multiple linear regression analyses, at FCz for the time windows − 100 to 300 and − 100 to 200 ms, respectively. Epochs were averaged for each participant, response type and motivational context (error, correct; gain, loss). Resulting *p* values were adjusted for multiple comparisons (Benjamini & Yekutieli, 2001).

## Results

### Behavioral analysis

**Fig. 2:**
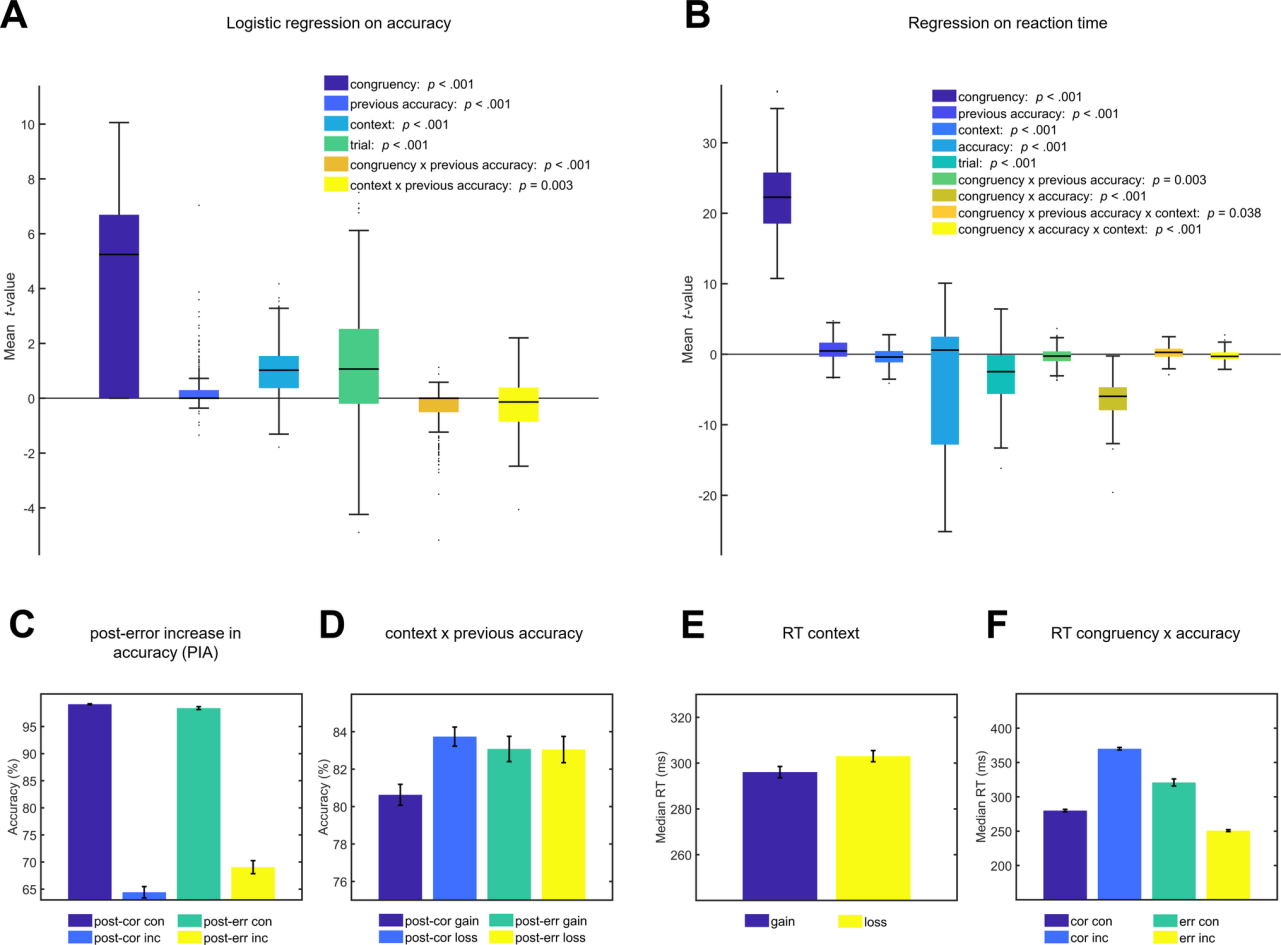
Visualization of behavioral results. Logistic regression on accuracy (**A**) and multiple single-trial regressions on RT (**B**) were used to assess behavioral effects in the task, controlling for confounding variables and the interdependence of effects. Interference effects are reduced after errors with regard to accuracy (**C**). Motivational context predicted RT, in that the loss context increased accuracy, as participants only showed post-error increase in accuracy (PIA) within the gain context (**D**), as well as RT (**E**). Stimulus incongruence decreased accuracy and increased RT, and interacted with accuracy (**F**). **A**,** B** depict mean within participant* t*-values, *p*-values are derived from t-tests of individual regression weights against zero and were Bonferroni corrected. RT is calculated as the mean of within-participants median RTs per condition. For **A**, **B**, boxes = interquartile range (IQR),—= median, whiskers = 1.5 × IQR, grey dots = outlier. For **C-F**, error bars represent the standard error of the mean (SEM) between subjects. See Table 1 for descriptive statistics of behavioral data. RT, reaction time; post-cor con, post-correct congruent trials; post-cor inc, post-correct incongruent trials; post-err con, post-error congruent trials; post-err inc, post-error incongruent trials; post-cor gain, post-correct gain trials; post-cor loss, post-correct loss trials; post-err gain, post-error gain trials; post-err loss = post-error loss trials; gain, gain trials; loss, loss trials; cor con, correct congruent trials; cor inc, correct incongruent trials; err con, error incongruent trials; err inc, error incongruent trials

Results were representative of flanker tasks and additionally reflected effects of motivational context (Fig. 2). Effects of interference were reflected in slower (*t*_220_ = 61.63, *p* < 0.001) and more erroneous (*t*_220_ = 20.64, *p* < 0.001) responses on incongruent trials. PES was reflected in a significant main effect for *previous accuracy* (*t*_220_ = 5.51, *p* < 0.001). Moreover, there was a general slowing in the loss compared with the gain context (*t*_220_ = − 5.06, *p* < 0.001). Additionally, PIA was present for the gain, but not for the loss context (Fig. 2d, interaction context*previous accuracy t_220_ = − 3.59, *p* = 0.003). Errors on the previous trial led to increased accuracy on incongruent, but not congruent trials (Fig. 2a, interaction congruency*previous accuracy t_220_ = − 7.03, *p* > 0.001). For more detailed information on the behavioral analyses see Supplement 3 and for descriptive RT analyses see Table S1. Impulsivity, compulsivity or their interaction did not significantly predict any of the behavioral measures, with all uncorrected *p* > 0.13 (PIA), *p* > 0.15 (PCA), *p* > 0.06 (PES), *p* > 0.13 (Error rate), and *p* > 0.33 (correct RT).

### EEG analysis

#### ERP analysis

**Fig. 3:**
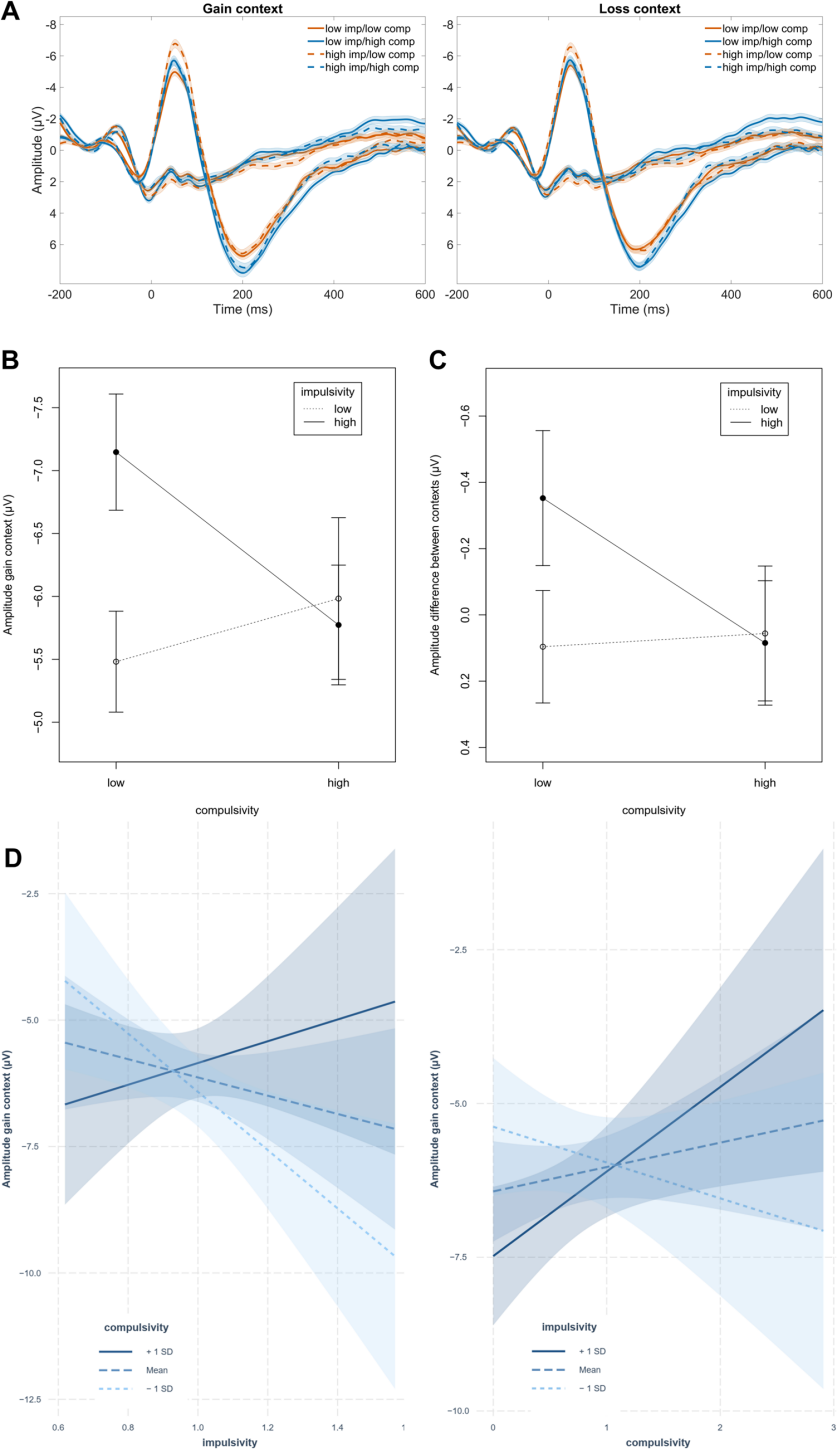
Visualization of response-locked ERPs for different levels of impulsivity and compulsivity. The sample was split by median for visualization purposes into: low impulsive/low compulsive, low impulsive/high compulsive, high impulsive/low compulsive, and high impulsive/high compulsive. (**A**) Time course of response-locked incongruent error and correct trials at mean amplitude of FCz and Cz, for the gain and the loss context. Shades indicate the SEM between subjects. (**B**) Visualization of the significant interaction of impulsivity and compulsivity in predicting the ERN amplitude at mean amplitude of FCz and Cz in the gain context. Error bars represent the SEM. (**C**) visualization of the significant interaction of impulsivity and compulsivity (see *EEG*_*error*_) in predicting the ERN amplitude difference between gain and loss context (ERN gain – ERN loss) at mean amplitude of FCz and Cz. Error bars represent the SEM between subjects. (**D**) Visualization of simple slopes for the interaction of impulsivity and compulsivity in the gain context*.* First panel, simple slopes for the association between impulsivity and ERN amplitude in the gain context for low (− 1 SD below the mean), moderate (mean), and high (+ 1 SD above the mean) levels of compulsivity. Second panel, simple slopes for the association between compulsivity and ERN amplitude in the gain context for low (− 1 SD below the mean), moderate (mean), and high (+ 1 SD above the mean) levels of impulsivity

Figure 3 depicts response-locked ERPs for different levels of impulsivity and compulsivity. All participants exhibited pronounced negativities following errors compared to correct responses, at frontocentral electrode sites. Within the gain context, the ERN amplitude was significantly predicted by impulsivity (*β* = − 7.21, *t* = − 2.70, *p*_*corrected*_ = 0.023, 95% confidence interval [CI] = [− 11.52, − 1.47]), compulsivity (*β* = − 6.22, *t* = − 2.88, *p*_*corrected*_ = 0.032, 95% CI = [− 11.18, − 0.35]), as well as their interaction (*β* = 6.71, *t* = 2.32, *p*_*corrected*_ = 0.032, 95% CI = [0.47, 11.34]).

Simple slopes for the association between impulsivity and ERN amplitude were tested for low, moderate, and high levels of compulsivity. This revealed a crossover interaction, as impulsivity was negatively associated with the ERN amplitude at low and moderate levels (predicting higher amplitudes), but positively (predicting lower amplitudes) at high levels of compulsivity; compulsivity was negatively associated with the ERN amplitude at low levels (predicting higher amplitudes), but positively (predicting lower amplitudes) at moderate and high levels of impulsivity. See Supplement 4 for more detailed information on simple slopes testing results. Figure 3d depicts the simple slopes for the interaction. The ERN was therefore elevated for both higher impulsivity and compulsivity when the other predictor was low, and highest for high impulsive but low compulsive individuals, see Fig. 3b for a visualization of this interaction. Within the loss context, there were no significant effects of impulsivity (*β* = − 4.81, *t* = − 0.77, *p*_*uncorrected*_ = 0.078, 95% CI = [− 10.14, 0.54]), compulsivity (*β* = − 4.17, *t* = − 1.43, *p*_*uncorrected*_ = 0.155, 95% CI = [− 9.91, 1.59]) or their interaction (*β* = 4.54, *t* = 1.55, *p*_*uncorrected*_ = 0.124, 95% CI = [− 1.25, 10.29]). There were no effects on early Pe, late Pe, or CRN in either motivational context. See Supplement 4 for additional analyses controlling for other subfacets of impulsivity as well as anxiety.

#### Single-trial analysis

**Fig. 4:**
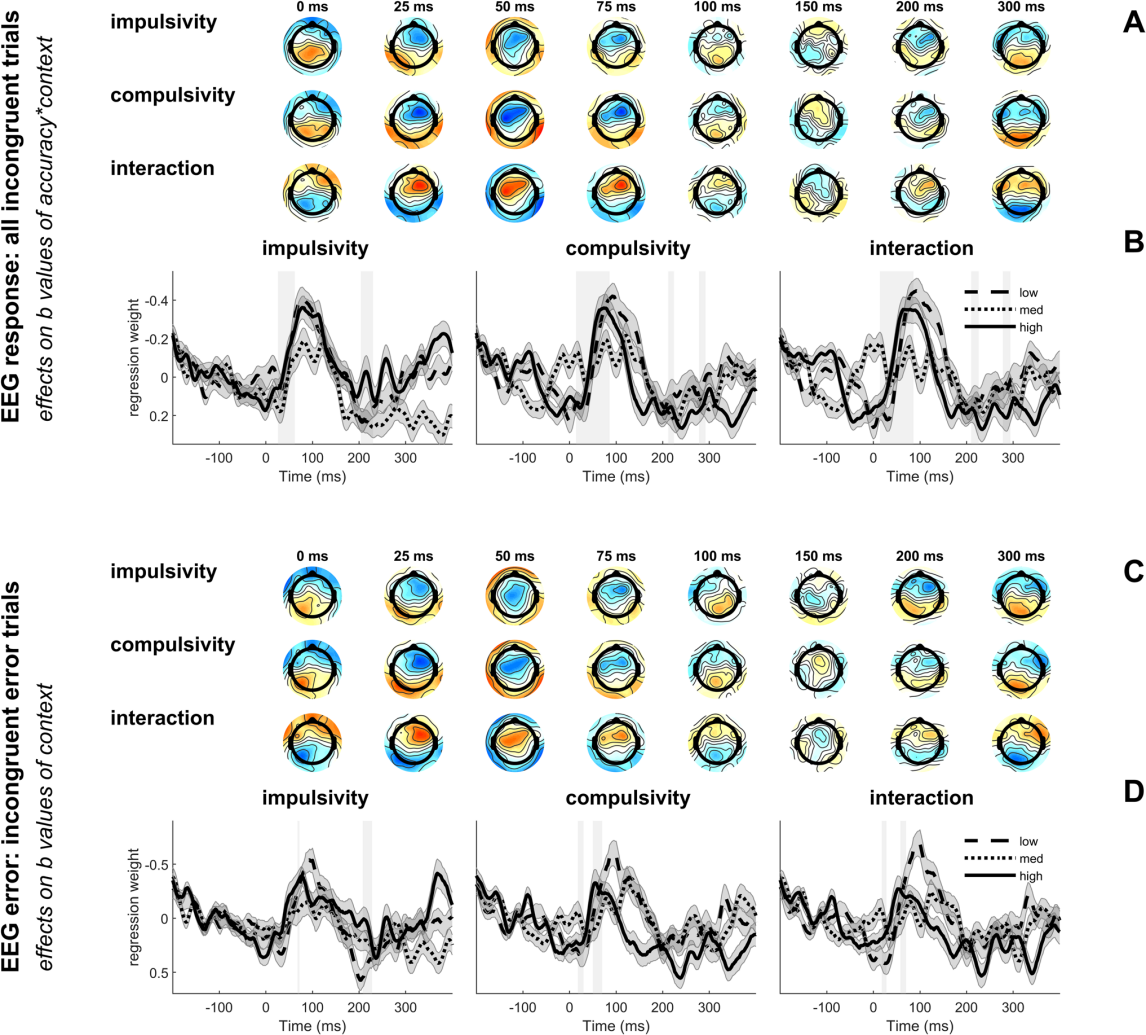
Visualization of response-locked single-trial regression analyses, depicting the influence of impulsivity, compulsivity, and their interaction on EEG activity. Results for the influence of the interaction of accuracy and motivational context on response processing *(EEG*_*response*_: **A**, **B**) and for the effect of motivational context on error processing *(EEG*_*error*_: **C**, **D**) are presented*.* Regression weights are used to quantify the extent to which the respective predictors explain variance in brain activity. For the interaction between impulsivity and compulsivity, the value of the regression weights quantifies their joint influence. Since the interaction variable is the product of the values of impulsivity and compulsivity, low (impulsivity*compulsivity = small value), medium (impulsivity*compulsivity = moderate value) and high values (impulsivity*compulsivity = large value) represent different combinations of the predictors, reflecting their combined effect on the outcome. (**A**) Time course of regression weights for regressors impulsivity, compulsivity and their interaction on the interaction between accuracy and context (accuracy*context) for the *EEG*_*response*_ model, visualized as time-resolved scalp topographies. (**B**) Time course of regression weights for impulsivity, compulsivity and their interaction (low, medium, and high values) on the interaction effect between accuracy and context (accuracy*context) at electrode FCz. Thick lines represent mean regression weights; shades indicate the SEM between subjects. Gray shading behind the waveforms indicate significance at *p* < 0.05/3. (**C**) Time course of topographical response-locked regression weights of impulsivity, compulsivity and their interaction on neural responses to motivational context (*context*) in the *EEG*_*error*_ model (including only incongruent errors). (**D**) Time course of regression weights for impulsivity, compulsivity and their interaction (low, medium, and high values) on the context effect at electrode FCz. As in panel B, thick lines represent mean regression weights; shades indicate the SEM between subjects. Gray shading behind the waveforms indicate significance at *p* < 0.05/3. *EEG*_*response*_*,* general linear model for response-locked brain activity (including incongruent trials); *EEG*_*error*_*,* general linear model for error-related brain activity (including incongruent trials).

We employed single-trial robust regression to obtain a regression weight time-course for performance monitoring-related EEG activity for all electrodes (Fischer & Ullsperger, 2013). As the second level model for incongruent trials (b values from *EEG*_*response*_) revealed significant effects of impulsivity (FCz: 26–62, 204–230 ms; Pz: none), compulsivity (FCz: 14–86, 212–224, 278–292 ms; Pz: none), and their interaction (FCz: 14–86, 210–226, 278–294 ms; Pz: none) for time windows corresponding to the ERN and early Pe for the interaction of accuracy*context (Figs. 4a, b), we investigated this further with the previously described follow-up models.

The model comparing incongruent error trials (*EEG*_*error*_) revealed no significant effect of context on the EEG at time windows corresponding to the ERN or Pe (similar to Overmeyer et al., 2023). However, there were significant effects of impulsivity (FCz: 68–72, 208–228 ms; Pz: 286–292 ms), compulsivity (FCz: 18–30, 50–70 ms; Pz: none), and their interaction (FCz: 14–28, 58–70 ms; Pz: none) for time windows corresponding to the ERN and early Pe (Figs. 4c, d) for this regressor (b values from *EEG*_*error*_). For both impulsivity and compulsivity, regression weights were more negative, indicating that higher levels were associated with smaller differences in ERN amplitudes between contexts, suggesting the ERN was less influenced by context. More positive regression weights for the interaction indicated larger differences between contexts if the interaction was low, which is exemplified by the difference between gain and loss context for low impulsive/low compulsive individuals (Fig. 4c). High impulsivity also predicted lower context differences for the early Pe. For regressor *accuracy* in the gain context (b values from *EEG*_*gain*_), impulsivity had no significant effect, whereas both compulsivity (FCz: 2–28 ms; Pz: 496–504 ms) and the interaction (FCz: 4–28 ms; Pz: 492–504 ms) were significant, with effects corresponding to the ERN and CRN time window (Figs. S3a, b). High compulsivity predicted larger differences between error and correct trials, and the interaction suggests that individuals exhibiting the highest difference were more impulsive and less compulsive, and individuals exhibiting the lowest difference scored lower on both. For regressor *accuracy* in the loss context (b values from *EEG*_*loss*_), impulsivity had a significant effect (FCz: none; Pz: 380–416, 450–466 ms), whereas both compulsivity and the interaction effects were not significant (Figs. S3c, d). For results of the first-level models see Supplement 4.

#### Time frequency analysis

**Fig. 5:**
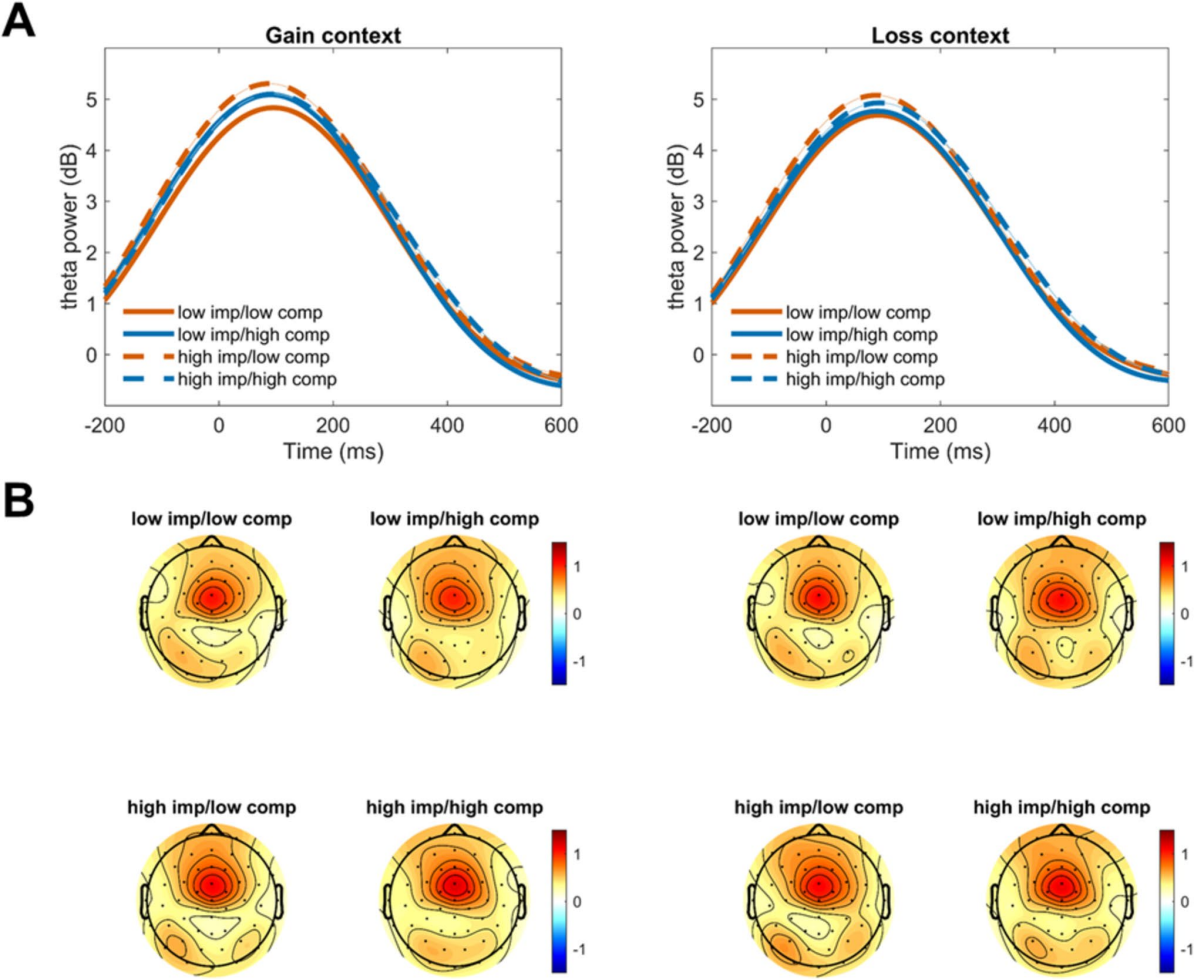
Visualization of response-locked time–frequency results. The sample was split by median for visualization purposes into: low impulsive/low compulsive, low impulsive/high compulsive, high impulsive/low compulsive, and high impulsive/high compulsive. (**A**) Time course of mean theta power (3–7 Hz) response-locked incongruent error trials at FCz, for the potential gain context and the loss avoidance context. (**B**) Topographical maps of mean response-locked theta activity of the sample split by median, for the gain context (left two columns) and the loss context (right two columns): low impulsive/low compulsive, low impulsive/high compulsive, high impulsive/low compulsive, and high impulsive/high compulsive; at 0–100 ms

Within either context, theta power was not significantly predicted by impulsivity, compulsivity, or their interaction (Fig. 5). However, there was a trend towards significance within the gain context (impulsivity: *β* = 1.78, *t* = 1.74, *p*_*corrected*_ = 0.083, 95% CI = [− 0.24, 3.81]; compulsivity: *β* = 2.11, *t* = 1.91, *p*_*corrected*_ = 0.083, 95% CI = [− 0.06, 4.29]; interaction: *β* = − 2.05, *t* = − 1.85, *p*_*corrected*_ = 0.083, 95% CI = [− 4.23, 0.14]) for error trials. There were also no significant effects for delta power or any of the combinations of response type and motivational context (all *p*_*corrected*_ > 0.10).

## Discussion

There is growing evidence that the interaction of impulsivity and compulsivity may be an important influence on cognitive and behavioral outcomes, particularly in the presence of motivational incentives (Dalgleish et al., 2020; Ferreira et al., 2021; Tiego et al., 2019). The shared variance between impulsivity and compulsivity was shown to predict co-occurring symptoms manifesting in maladaptive behavior, highlighting the value of examining the neural underpinnings of these processes transdiagnostically (Tiego et al., 2019). This study investigated how performance monitoring relates to impulsivity and compulsivity, aiming to disentangle motivational effects of potential gain and loss avoidance. Reward processing is especially relevant to maladaptive behavior associated with impulsivity and compulsivity, such as substance use, gambling, and eating (Albertella et al., 2019; Dawe et al., 2004; Yucel et al., 2019). Impulsivity, compulsivity and their interaction showed significant effects on error-related brain activity in trials with potential gain, but not in loss-avoidance trials. Consistent with expectations, higher levels of compulsivity were associated with larger ERN amplitudes, and at trend level to theta power. While the direction of effects is similar, the observed differences in impulsivity and compulsivity effects on ERN and theta power may reflect overlapping but partially distinct neural mechanisms captured by these measures, because theta power has additionally been linked to cognitive control engagement (Eisma et al., 2021; Senoussi et al., 2022).

However, contrary to expectations, impulsivity was also positively associated with ERN amplitude, and at trend level with theta power. Importantly, an interaction between impulsivity and compulsivity was observed in predicting the ERN amplitude in the gain context: individuals with high impulsivity and low compulsivity exhibited the highest amplitude, whereas individuals scoring low on both dimensions exhibited the lowest amplitudes. Differences in ERN amplitude between gain and loss were most pronounced when impulsivity was high and compulsivity was low. In contrast, these differences were less pronounced when compulsivity was high, suggesting reduced flexibility in performance monitoring between contexts (Endrass et al., 2010; Hajcak et al., 2005). Thus, results indicate that motivational context exerts a stronger influence on individuals with high impulsivity compared with those with high compulsivity, aligning with our expectations. Additionally, the amplitude difference between error and correct trials was larger in individuals with high compulsivity, and for individuals with high impulsivity when compulsivity was low.

We also observed an effect of impulsivity on the early Pe, indicating that high impulsivity was associated with reduced differences in Pe amplitude across contexts and therefore smaller contextual differences in error evaluation and awareness (Ullsperger et al., 2014a, 2014b). This aligns with previous work linking impulsivity to lower error awareness (Dali et al., 2023; O’Connell et al., 2009) and is especially interesting when considering the potential relevance of error awareness for behavioral adaptation (Orr & Carrasco, 2011). No significant effects of either predictor or their interaction were observed for other ERP measures. Furthermore, while our results aligned with previous studies reporting behavioral differences between motivational contexts (Fahey et al., 2025; Overmeyer et al., 2023; Yee et al., 2021), we did not observe any effects of compulsivity or impulsivity on behavioral measures, consistent with other studies (Gründler et al., 2009; Hajcak & Simons, 2002; Hill et al., 2016). This suggests that neural differences are likely driven by differences in impulsivity and compulsivity rather than being behaviorally mediated.

Increased motivational salience of errors is commonly associated with larger ERN amplitudes, whereas motivational deficits and poor task engagement are associated with reduced ERN amplitudes (Hajcak et al., 2005; Ullsperger et al., 2014a, 2014b; Weinberg et al., 2012). In our study high compulsivity was associated with higher ERN amplitudes; this was, however, only true for the gain context and was influenced by an interaction with impulsivity. These findings align with prior research linking high compulsivity and OCD to enhanced ERN amplitudes (Bellato et al., 2021; Endrass et al., 2008, 2010). However, studies examining compulsivity within motivational contexts remain limited. In a study with monetary losses, gains and a neutral condition, ERN amplitudes were found to be elevated in individuals with OCD; however, analyses were performed across combined motivational conditions, potentially masking differential effects for the three motivational contexts (Stern et al., 2010). Similarly, Ruchsow et al. (2005) reported enhanced ERN amplitudes in OCD patients but did not disentangle the effects of gains versus losses. Our observation that compulsivity did not affect the ERN within loss-avoidance trials and that the difference between potential gain and loss-avoidance trials was smaller the higher individuals scored on compulsivity aligns with results from a previous study reporting diminished effects of error significance for individuals with OCD (Endrass et al., 2010). For OCD patients, there was no difference in ERN amplitude between a neutral and a punishment condition, whereas healthy controls exhibited an amplitude enhancement between neutral and punishment condition. Interestingly, the patient group had significantly larger ERN amplitudes in the neutral condition, while there was no difference between groups within the punishment condition. A study focusing on conscientiousness, a construct related to compulsivity (Samuel & Widiger, 2011), also observed smaller motivation-related changes in ERN amplitude (Pailing & Segalowitz, 2004). Analogue to conclusions drawn by Endrass et al. (2010), this may suggest that individuals scoring high on compulsivity are unable to adaptively regulate their monitoring activity, independent of motivational context. These findings highlight the importance of including neutral conditions to control for amplitude changes in contrast to a condition with no manipulations of error value.

Impulsivity was also associated with elevated ERN amplitudes in the gain context and showed no association with loss avoidance, contrary to expectations. Furthermore, high impulsivity was associated with reduced differentiation between contexts for the ERN and early Pe, with this effect being influenced by an interaction with compulsivity. Previous research on the association of impulsivity with motivational context is sparse and inconsistent. While only one study directly investigated the interplay between impulsivity and motivational effects on the ERN, its findings revealed no difference between low and highly impulsive individuals for reward-motivated trials, but it identified attenuated ERN on punishment-motivated trials for high vs. low impulsive individuals (Potts et al., 2006). Notably, these findings are inconsistent with our results. Discrepancies may reflect variability in sample sizes, as robust assessments of individual differences require large samples to avoid overestimating effects (Button et al., 2013; Larson & Carbine, 2017). For example, two recent studies with larger samples failed to replicate the previously reported association of impulsivity with attenuated ERN amplitudes in standard tasks without motivational incentives (Bernoster et al., 2019; Overmeyer & Endrass, 2024). By explicitly controlling for substance use in our sample, we may also have reduced relevant variability in impulsivity, leading to different results. Findings for individuals with a current SUD or other externalizing diagnoses robustly show attenuated ERN amplitudes, mostly on standard tasks without external motivational context (for an overview see Pasion & Barbosa, 2019; Weinberg et al., 2015). However, it has been proposed that these effects may be attributed to the influence of current substance use (Gorka et al., 2019). Alternatively, results may also suggest that impairments in cognitive and motivational processes required for implementing adaptive behavior may only emerge at later stages, and not during error monitoring. For example, impulsivity was associated with deficits in reward-based decision-making only in tasks involving learning, indicating a decreased ability to adapt behavior in response to fluctuations in motivational significance (Franken et al., 2008). Similarly, high impulsivity was shown to predict reduced goal-directed control in a devaluation paradigm (Hogarth et al., 2012).

Elevated ERN amplitudes for high impulsivity in the potential gain context may also indicate higher subjective error significance (Endrass et al., 2010; Hajcak et al., 2005). This could be connected to higher reward responsiveness, which is distinct from impulsivity, but has been related to impulsivity (Dawe et al., 2004; Martin & Potts, 2004). Interestingly, results on equivalent ERN amplitude across motivational contexts for highly impulsive individuals and smokers (Potts et al., 2006, 2014) are consistent with our data and also suggest diminished ability to adaptively regulate performance monitoring according to external demands, an effect likely influenced by compulsivity. Importantly, as impulsivity is a multidimensional construct, it seems likely that subfacets of impulsivity may have differential associations with different measures of the ERN and connections may depend on interactions with other traits (Hill et al., 2016), which is exemplified by the significant interactions between impulsivity and compulsivity within our sample. For example, we observed that lack of premeditation, a subfacet of impulsivity assessed by the UPPS Impulsive Behavior Scale (Schmidt et al., 2008; Whiteside & Lynam, 2001) predicted attenuated ERN amplitudes within the potential gain context (see Supplement 4). For the loss avoidance context, lack of premeditation and in addition sensation seeking predicted attenuated ERN amplitudes. This is partially consistent with prior research (Michałowski et al., 2020; Santesso & Segalowitz, 2009; Zheng et al., 2014) using standard tasks (but see Bernoster et al., 2019; Hill et al., 2016; Vocat et al., 2008), highlighting the complexity of these relationships. These differential associations and potential interactions may also help to explain inconsistencies in the literature.

Crucially, we observed interaction effects between impulsivity and compulsivity. Specifically, high impulsivity predicted larger ERN amplitudes in the potential gain context only for lower compulsivity, and ERN amplitudes were smallest when both impulsivity and compulsivity were low. Additionally, the regression-based differentiation between gain and loss trials within the ERN time window was more pronounced when compulsivity was low, and most pronounced when impulsivity was high and compulsivity was low. Individuals scoring low on compulsivity may therefore display more adaptive modulation of their monitoring, according to error significance.

Limitations of the current study include the absence of a non-incentive context in the task. There is evidence that ERPs during response processing are sensitive to incentive contexts as compared to nonincentive ones (Endrass et al., 2010; Maruo et al., 2017). Aside from that, context effects might be obscured in tasks with a trial-by-trial compared with a block-wise manipulation because the task requires constant attention (Tullo et al., 2020). Additionally, we controlled for use of illicit substances, which may have restricted the range of impulsivity or subfacets of impulsivity within our sample. However, participants exhibited a broad range of self-reported impulsivity on the BIS-11 scale. We also utilized only one scale to measure each construct within our main analyses, which may limit the generalizability of our findings to other operationalizations of impulsivity and compulsivity. Future research should incorporate multiple feedback manipulations, additional scales assessing impulsivity and compulsivity and include participants using different substances to improve generalizability.

## Conclusions

The present study demonstrates that both impulsivity and compulsivity are associated with deficits in adaptive regulation of performance monitoring across different motivational contexts. High impulsivity and compulsivity predicted elevated ERN amplitudes, and on a trend level elevated theta power, within potential gain trials, suggesting heightened subjective error significance (Endrass et al., 2010; Hajcak et al., 2005). By establishing an association of both constructs to heightened significance of errors in the face of potential rewards as well as deficits in adaptive regulation across different motivational contexts, our findings suggest that impulsivity and compulsivity may influence how errors are processed in different motivational environments and raise the question of how this may affect decision-making and the employment of cognitive control. Future studies should include neutral conditions or standard tasks to help elucidate how error monitoring changes with and without external value manipulations. Considering interactions between transdiagnostic traits may aid in understanding the complex dynamics emerging when examining motivational effects in the investigation of neural correlates of error processing.

## Supplementary Information

Below is the link to the electronic supplementary material.

Supplementary file1 (PDF 1.54 MB)

## Data Availability

The data that support the findings of this study are available on The Open Science Framework under https://osf.io/asbzm/.

## Code availability

The code associated with this research paper is made available on the OSF for the purpose of transparency and reproducibility. The repository can be accessed here: https://osf.io/asbzm/. Code used for the single-trial analyses can be found at http://adrianfischer.de/teaching.html.
