# Supplementary material for: The impact of impulsivity and compulsivity on error processing in different motivational contexts
Source: Cogn Affect Behav Neurosci. 2025 Mar 5;25(4):952–70. doi: 10.3758/s13415-025-01281-5 (PMC12356756; doi:10.3758/s13415-025-01281-5)
Supplement: Supplementary file 1 — Supplementary file1 (PDF 1.54 MB) [file 13415_2025_1281_MOESM1_ESM.pdf]

Supplementary Note

The impact of impulsivity and compulsivity on error processing in different motivational contexts

1. Additional information on recruitment

We recruited 253 participants from the general population in the Dresden area targeting impulsivity and compulsivity dimensions based on pre-study scores from the Obsessive-Compulsive Inventory-Revised (OCI-R; Foa et al., 2002; Gönner et al., 2007b) and the Barratt Impulsiveness Scale (BIS-11; Patton et al., 1995).

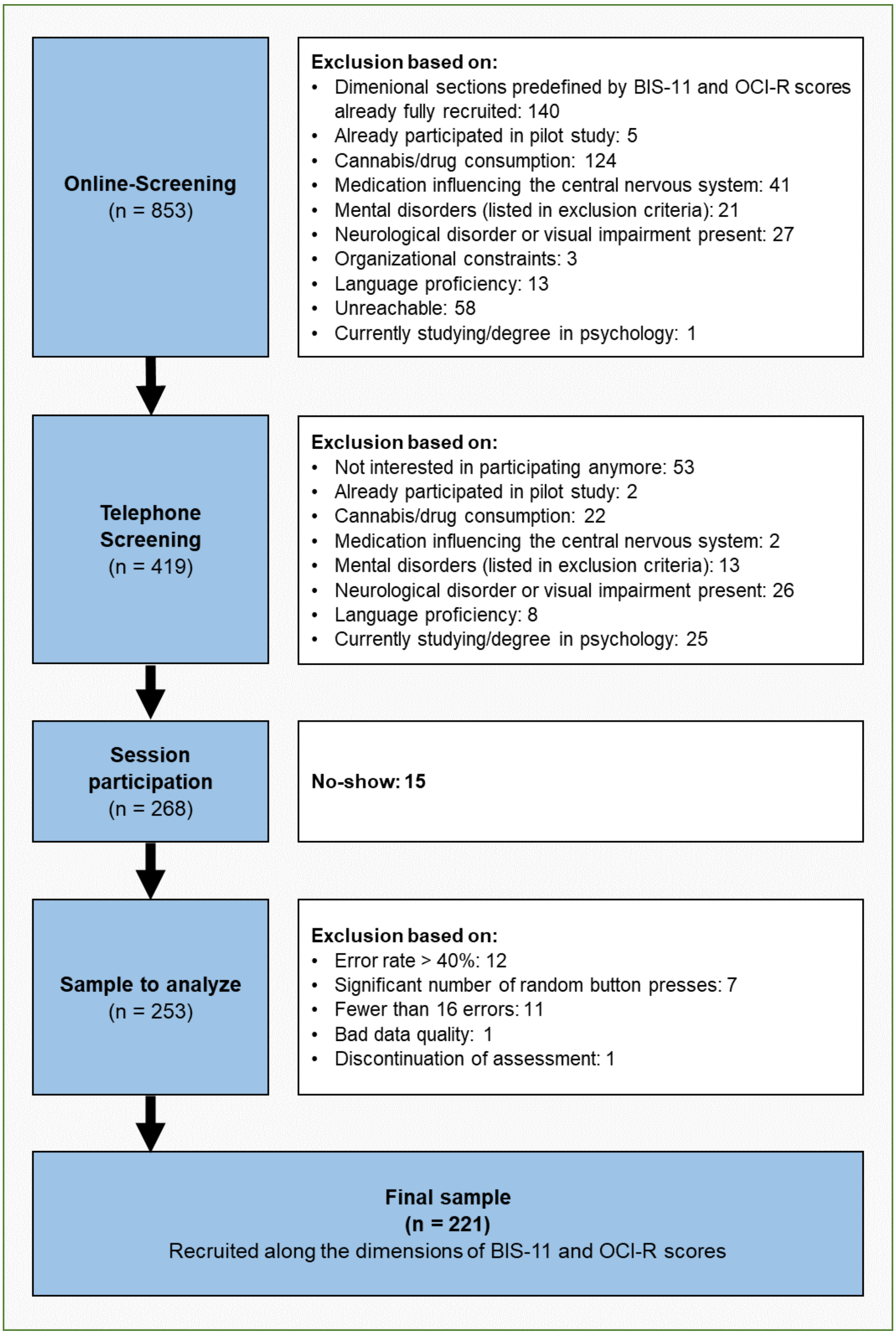

Fig. S1. *Schematic depiction of recruitment.* BIS-11, Barratt Impulsiveness Scale; OCI-R, Obsessive-Compulsive Inventory-Revised.

Participants scoring below 10 on the OCI-R were categorized as low compulsive, those scoring above 13 as high compulsive, and those in-between as medium compulsive (Abramowitz & Deacon, 2006). Similarly, BIS-11 scores below 57 were classified as low impulsive, scores above 67 as high impulsive, and scores in-between as medium impulsive, based on normative population data (Stanford et al., 2009).

Initially, participants were recruited as a convenience sample. After recruiting half of the pre-defined sample, we shifted to stratified sampling, evenly targeting sections formed by the intersection of tertiles within the impulsivity-compulsivity dimensional space defined by the OCI-R and BIS-11. We screened 853 participants using an online survey for inclusion and exclusion criteria and conducted a telephone interview with 419 participants (see Figure S1 for a schematic depiction of recruitment). Age and gender distributions were paralleled within the sample. Consequently, there was no correlation between impulsivity and compulsivity in this sample (r = -0.005, p = 0.945).

2. Additional information on used R packages

All analyses were carried out with MATLAB (The MathWorks Inc., 2018) and R (R Core Team, 2022), using the packages readxl (Wickham & Bryan, 2022), dplyr (Wickham et al., 2022), interactions (Long, 2022) as well as a function for an interaction plot (Baguley, 2010).

3. Behavioral effects

We employed two multiple robust regression models onto each participant's single-trial accuracy and log-transformed reaction time (RT) to determine predictors of influence (Fischer et al., 2018). The logistic accuracy model (GLM1) was specified by: $accuracy = b_0 + congruency \times b_1 + previous\ accuracy \times b_2 + context \times b_3 + trial \times b_4 + e$. The predictors are: *congruency* (congruency between flanker and target; -1 = congruent, 1 = incongruent), *previous accuracy* (accuracy of the immediately preceding trial; -1 = correct, 1 = error), *context* (incentive context of the current trial; -1 = loss, 1 = gain), *accuracy* (of the current trial; -1 = correct, 1 = error) and *trial* (trial number, reflecting the time on the task). The RT model (GLM2) was specified as follows: $log(RT) = b_0 + congruency \times b_1 + previous\ accuracy \times b_2 + context \times b_3 + accuracy \times b_4 + trial \times b_5 + e$. *Trial* mainly served to control for unspecific effects of task duration (e.g. adjustments of speed-accuracy trade-offs over the task or blocks, or fatigue). Individual t-values per regressor were tested via two-sided t-tests against zero, on group level, all p-values were Bonferroni corrected.

Behavioral effects in the current study match those typically observed in a flanker task. On incongruent trials, participants were significantly less accurate (main effect *congruency*, t_{220} = 20.64, p < .001 two-sided t-test against zero, all p-values are Bonferroni corrected). Accuracy was also lower in the gain context (main effect *context*, t_{220} = 14.10, p < .001). After error commission, accuracy increased on the following trial (post-error increase in accuracy, main effect of *previous accuracy*, t_{220} = 6.01, p < .001). This effect was modulated by congruency of the current trial (*congruency*previous accuracy*, t_{220} = -7.03, p < .001), as well as context of the current trial (*context*previous accuracy*, t_{220} = -3.59, p = 0.003).

RT was lower on congruent trials (main effect *congruency*, t_{220} = 61.63, p < .001), confirming the presence of an interference effect (Cohen et al., 2000). RT was higher for the loss context

(main effect *context*, t_{220} = -5.06, p < .001). Error RTs were faster than correct RTs (main effect of *accuracy*, t_{220} = -5.96, p = < .001). This was modulated by congruency (*congruency*accuracy*, t_{129} = -30.18, p < .001) and context (*congruency*accuracy*context*, t_{220} = -4.18, p < .001). Following error commission, RTs were slower (main effect *previous accuracy*, t_{220} = 5.51, p < .001), confirming post-error slowing (PES). Post-error slowing (PES) was modulated by motivational context and congruency (*congruency*previous accuracy*, t_{220} = -3.66, p = 0.003, *congruency*previous accuracy*context*, t_{220} = 2.89, p = 0.038).

See Table S1 for descriptive statistics of behavioral data.

Table S1

Task performance and measures of performance monitoring (means and standard deviations) in the Monetary Incentive Flanker Task.

	**All trials**		**Gain trials**		**Loss trials**	
	M	*SD*	*M*	*SD*	*M*	*SD*
Correct trials (ms)						
Correct incongruent RT	375	32	376	33	375	31
Correct congruent RT	285	29	284	29	287	29
Trials around errors (ms)						
Error RT	255	26	254	27	257	27
Post-correct RT	318	33	318	33	320	32
Post-error RT	324	36	320	37	327	37
Pre-error RT	304	31	302	31	305	33
Accuracy (%)						
Error rate	17.74	7.88	19.03	8.17	16.44	7.87
PEA	85.63	8.77	85.55	9.24	85.68	9.47
PCA	84.98	7.14	83.62	7.66	86.29	6.93

Note. RT, reaction time; Error reaction times refer to all error trials; Pre-error reaction times refer to correct trials before error commission; Post-error reaction times refer to correct trials after error commission; Post-correct reaction times refer to correct trials after correct responses; PEA, Post Error Accuracy; PCA, Post Correct Accuracy; Gain trials, context of current trial is potential gain context; Loss trials, context of current trial is loss avoidance context.

4. EEG analysis

Additional ERP analysis results. See Figure S2 for a visualization of the ERN and CRN in the gain and loss context, averaged for the complete sample. Simple slopes for the association between impulsivity and ERN amplitude in the gain context were tested for low (-1 SD below the mean), moderate (mean), and high (+1 SD above the mean) levels of compulsivity. This revealed a crossover interaction, as impulsivity was negatively associated with the ERN amplitude at low (β = -5.76, *SE* = 2.22, t = -2.59, p = .010) and moderate (β = -1.81, *SE* = 1.70, t = -1.06, p = .290) levels, but positively at high levels of compulsivity (β = 2.15, *SE* = 2.58, t = 0.83, p = .410). Compulsivity was negatively associated with the ERN amplitude at low (β = -.58, *SE* = .59, t = -.99, p = .320) levels, but positively at moderate (β = .40, *SE* = .42, t = .96, p = .340) and high levels of impulsivity (β = 1.37, *SE* = 0.60, t = 2.30, p = .020). Figure 4d depicts the simple slopes for the interaction.

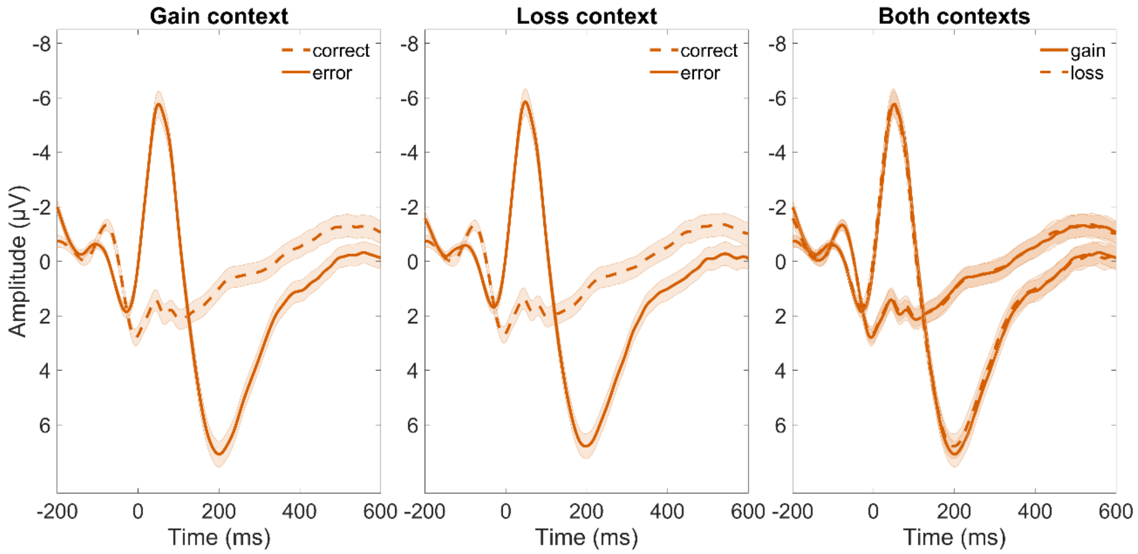

Fig. S2. *Visualization of response-locked ERPs for different motivational contexts*. Time course of response-locked incongruent error and correct trials at mean amplitude of FCz and Cz, for the gain and the loss context, as well as error and correct trials for both contexts. Shades indicate the SEM between subjects.

We also performed an explorative step-wise regression on the ERN including the subscales of the UPPS Impulsive Behavior Scale (urgency, lack of premeditation, lack of perseverance, sensation seeking; Schmidt et al., 2008) as well as the as the anxiety subscale of the Depression Anxiety Stress Scales (Henry & Crawford, 2005; Nilges & Essau, 2015) and both trait subscales of the State-Trait Inventory for Cognitive and Somatic Anxiety (STICSA; Overmeyer & Endrass, 2023; Ree et al., 2008) as additional predictors to impulsivity as measured by the Barratt Impulsiveness Scale (BIS; Patton et al., 1995; Stanford et al., 2009), compulsivity as measured by the Obsessive-Compulsive Inventory-Revised (OCI; Foa et al., 2002; Gönner et al., 2007a), and their interaction. In addition to subfacets of impulsivity potentially contributing differently to the ERN (Santesso & Segalowitz, 2009; Zheng et al., 2014), anxiety has repeatedly been linked to ERN amplitude (e.g. Klawohn et al., 2020; Zambrano-Vazquez & Allen, 2014). For the gain context, the backward step-wise regression model, using the AIC as a threshold, reduced the predictors from 10 to 4: impulsivity (BIS; $\beta = -0.16$, $t = -3.31$, $p_{uncorrected} = .001$), compulsivity (OCI; $\beta = -0.35$, $t = -1.90$, $p_{uncorrected} = .059$), the interaction of impulsivity (BIS) and compulsivity (OCI; $\beta = 6.27$, $t = 2.19$, $p_{uncorrected} = .030$), and the subscale lack of premeditation from the UPPS ($\beta = 0.15$, $t = 2.04$, $p_{uncorrected} = .043$). For the loss context, the backward step-wise regression model, using the AIC as a threshold, also reduced the predictors from 10 to 4: impulsivity (BIS; $\beta = -5.27$, $t = -2.36$, $p_{uncorrected} = .019$), the interaction of impulsivity (BIS) and compulsivity (OCI; $\beta = 0.71$, $t = 1.57$, $p_{uncorrected} = .117$), and the subscales lack of premeditation ($\beta = 3.35$, $t = 1.86$, $p_{uncorrected} = .065$) and sensation seeking ($\beta = 1.9$, $t = 1.53$, $p_{uncorrected} = .128$) from the UPPS.

Additional single-trial analysis results. We employed single-trial robust regression to obtain a regression weight time-course for PM-related EEG activity for all electrodes (Fischer & Ullsperger, 2013). Additional models following up the main analysis including all incongruent trials (with regressors accuracy, context, accuracy*context, log scaled RT of the current trial,

response hand; $EEG_{response}$) were as follows: 1) a model including incongruent error trials (with regressors context, log scaled RT of the current trial, response hand; EEG_{error}) 2) a model including incongruent trials in the gain context (with regressors accuracy, log scaled RT of the current trial, response hand; EEG_{gain}), and 3) a model including incongruent trials in the loss context (with regressors accuracy, log scaled RT of the current trial, response hand; EEG_{loss}).

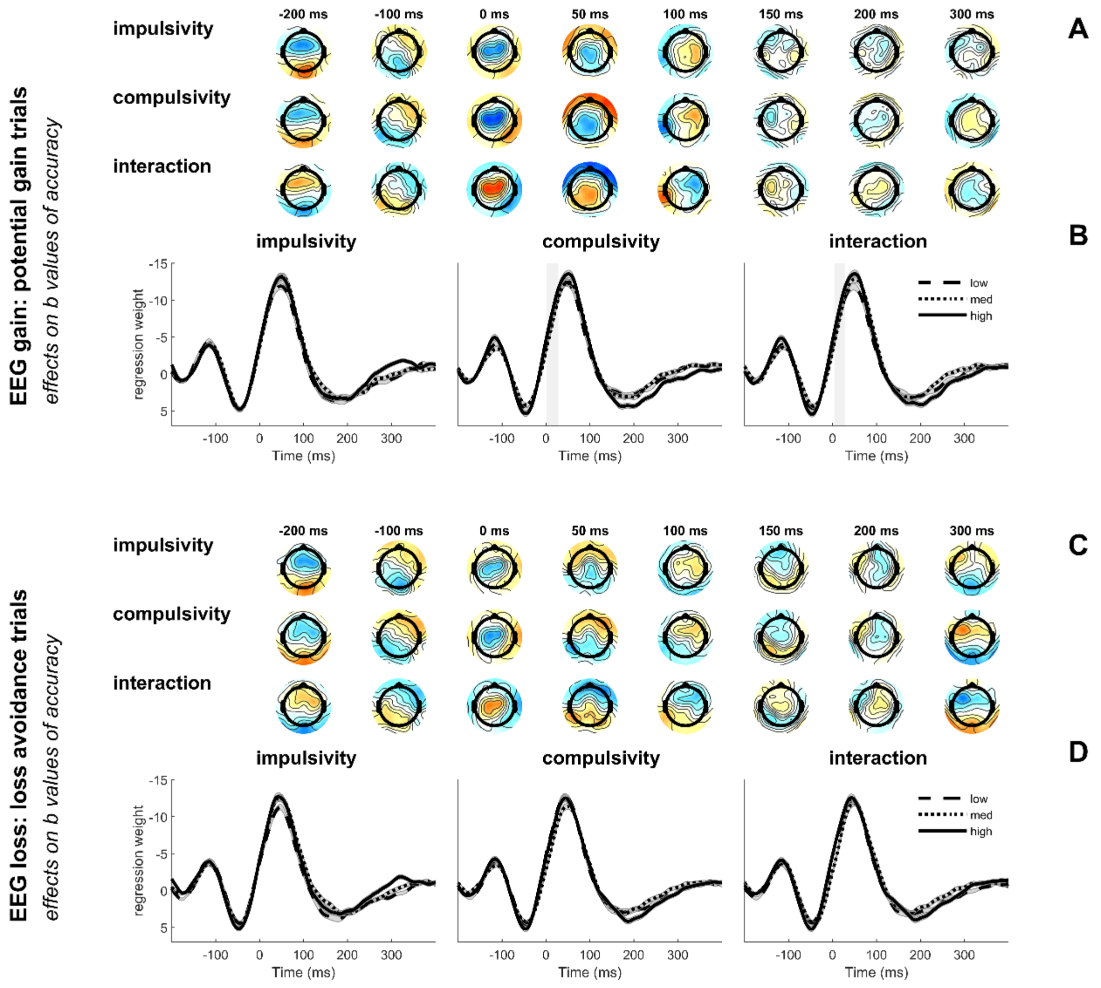

Fig. S3. *Visualization of response-locked single-trial analysis results.* **A**, time course of topographical response-locked regression effects for regressors impulsivity, compulsivity and their interaction on the effect of accuracy for the model including only potential gain trials (EEG_{gain}). **B**, time course of regression weights of the effect of accuracy for the model including only gain trials for impulsivity, compulsivity and their interaction (low, medium, and high, respectively), shown at electrode FCz. Thick lines, mean regression weights; shades indicate the SEM between subjects. Gray shading behind the waveforms indicate significance at $p < 0.05/3$. **C**, time course of topographical response-locked regression effects for regressors impulsivity, compulsivity and their interaction on the effect of accuracy for the model including only loss avoidance trials (EEG_{loss}). **D**, time course of regression weights of the effect of accuracy for the model including only loss trials for impulsivity, compulsivity and their interaction (low, medium, and high, respectively), shown at electrode FCz. Thick lines, mean regression weights; shades indicate the SEM between subjects. Gray shading behind the waveforms indicate significance at $p < 0.05/3$.

$EEG_{response}$ revealed significant effects of accuracy (FCz: 0-114, 128-274, 324-600 ms; Pz: 0-94, 108-600 ms), context (FCz: 0-36, 234-240 ms; Pz: none), and accuracy*context (FCz: 62-120 ms; Pz: none). For EEG_{error} there were no significant effects of context in predicting the EEG

(FCz: none; Pz: none). There were significant effects of accuracy for both EEG_{gain} (FCz: 0-114, 128-264, 328-600 ms; Pz: 0-94, 106-600 ms) and EEG_{loss} (FCz: 0-114, 128-276, 330-600 ms; Pz: 0-94, 108-600 ms). See figure S3 for a visualization of second-level results for EEG_{gain} and EEG_{loss}. We additionally performed a second level analysis on the b values of the *accuracy*context* regressor from $EEG_{response}$. Effects of impulsivity, compulsivity and their interaction remained consistent at FCz (impulsivity: 38-56 ms; compulsivity: 14-84 ms; impulsivity*compulsivity: 14-36, 38-40, 44-86 ms) with a p value of 0.05/3 (powered for 3 predictors) when controlling for age and sex. This was similar when adding anxiety to the model as measured using the DASS-21 anxiety scale (Henry & Crawford, 2005; Nilges & Essau, 2015). Neither age, sex nor anxiety exhibited significant effects on b values of *accuracy*context* at FCz.

5. Supplementary analysis of feedback effects

Single-trial analysis. We built a single-trial regression model for feedback-locked EEG following correct responses. The model included a regressor coding the feedback type of the current trial (positive/negative), the context of the current trial (gain, loss) and their interaction: *EEG = b0 + feedback type x b1 + context x b2 + feedback type*context x b3 + e*. We then conducted multiple linear regression analyses to compute the effects of impulsivity, compulsivity and their interaction (regressors were scaled) on standardized b values of the interaction regressor (*feedback type*context)* for the feedback-locked model, for frontocentral and parietal electrodes. Two follow-up models were built: a model on correct trials with negative feedback (with regressor context: $EEG_{negative\ feedback} = b0 + context \times b1 + e$), and a model on correct trials with positive feedback (with regressor context: $EEG_{positive\ feedback} = b0 + context \times b1 + e$). The significance threshold was corrected using a p value of 0.05/3.

The second-level model on the effect of context revealed significant effects of impulsivity (FCz: 240-244 ms; Pz: none), compulsivity (FCz: 234-250 ms; Pz: none), and their interaction (FCz: 232-252 ms; Pz: none) for time windows corresponding to the feedback-related negativity (FRN). We also investigated effects of context further with the previously described additional models. The model comparing correct trials with positive feedback between contexts revealed significant effects of impulsivity (FCz: 172-186, 398-400 ms; Pz: none), compulsivity (FCz: 166-190, 238-252, 274-290, 336-350, 392-412 ms; Pz: none), and their interaction (FCz: 166-190, 236-254, 272-296, 328-354, 394-410 ms; Pz: none) for time windows corresponding to the P2, FRN and P3a (see Figure S4a,b). The model comparing correct trials with negative feedback between contexts revealed no significant effects of impulsivity, compulsivity or their interaction (see Figure S4c,d).

ERP analysis. As the single-trial regression analyses examines contrasts between different conditions, we conducted an additional ERP analysis to specifically examine feedback-related ERPs. The FRN was determined as the mean activity within 240 to 340 ms (see Stewardson & Sambrook, 2023) in a cluster of frontocentral electrodes FCz and Cz. The P3a was defined as the mean activity between 325 and 425 ms after feedback onset in a cluster of frontocentral electrodes FCz and Cz. The P3b was defined as the mean activity between 350 and 600 ms after feedback onset in a cluster of parietal electrodes CPz and Pz. The effect of impulsivity and compulsivity and their interaction (regressors were scaled) was tested using multiple linear regression analyses, separately for context and feedback type. The resulting *p* values were

adjusted for multiple comparisons using the FDR procedure proposed by Benjamini and Yekutieli (2001).

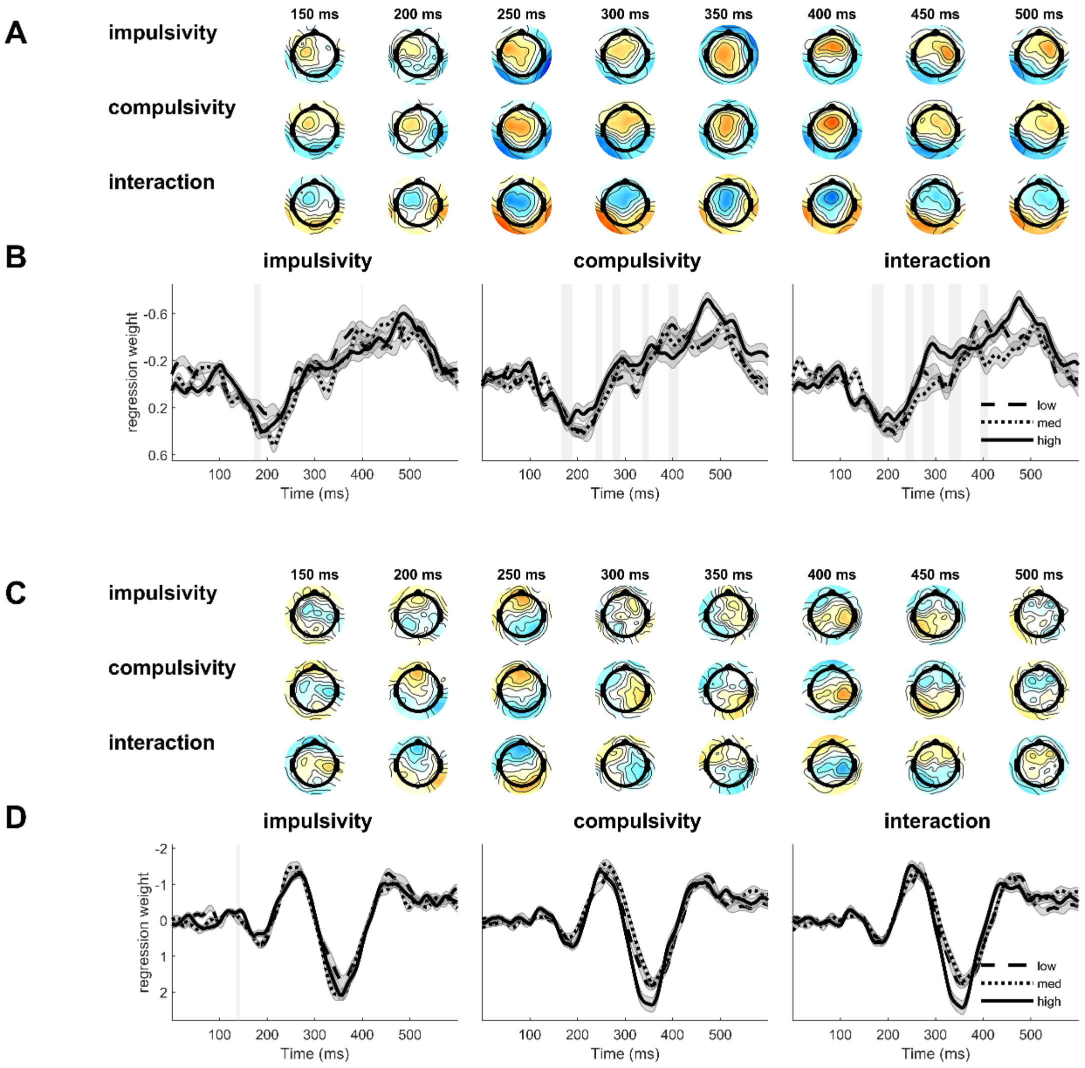

Fig. S4. *Visualization of feedback-locked single-trial analysis results.* **A**, time course of topographical feedback-locked regression effects for regressors impulsivity, compulsivity and their interaction on the effect of context for the model including only trials with POSITIVE FEEDBACK. **B**, time course of regression weights of the effect of context for the model including only trials with positive feedback for impulsivity, compulsivity and their interaction (low, medium, and high, respectively), shown at electrode FCz. Thick lines, mean regression weights; shades indicate the SEM between subjects. Gray shading behind the waveforms indicate significance at $p < 0.05/3$. **C**, time course of topographical feedback-locked regression effects for regressors impulsivity, compulsivity and their interaction on the effect of context for the model including only trials with NEGATIVE FEEDBACK. **D**, time course of regression weights of the effect of context for the model including only trials with negative feedback for impulsivity, compulsivity and their interaction (low, medium, and high, respectively), shown at electrode FCz. Thick lines, mean regression weights; shades indicate the SEM between subjects. Gray shading behind the waveforms indicate significance at $p < 0.05/3$.

Figure S5 and S6 present feedback-locked ERPs for different levels of impulsivity and compulsivity. All participants exhibited pronounced negativities following negative feedback compared to positive feedback, at frontocentral electrode sites. For positive feedback (see Figure S5), compulsivity and the interaction between impulsivity and compulsivity predicted the FRN

within the gain context on a trend level (compulsivity: β = 3.62, t = 2.00, $p_{corrected}$ =.077; interaction: β = -3.56, t = -1.96, $p_{corrected}$ =.077). We also observed significant effects of compulsivity and the interaction of impulsivity and compulsivity on the P3a (compulsivity: β = 4.30, t = 2.63, $p_{corrected}$ =.014; interaction: β = -4.37, t = -2.66, $p_{corrected}$ =.014) and the P3b (compulsivity: β = 3.16, t = 2.17, $p_{corrected}$ =.046; interaction: β = -3.21, t = -2.20, $p_{corrected}$ =.046). We did not observe any effect for the FRN or the P3a for positive feedback within the loss context (all p > .14). However, there was a trend for effects of compulsivity and the interaction between impulsivity and compulsivity on the P3b (compulsivity: β = 3.20, t = 2.07, $p_{corrected}$ =.069; interaction: β = -3.11, t = -2.01, $p_{corrected}$ =.069). For negative feedback (see Figure S6), there were no effects on the FRN, the P3a or the P3b for either context (all $p_{corrected}$ > .09).

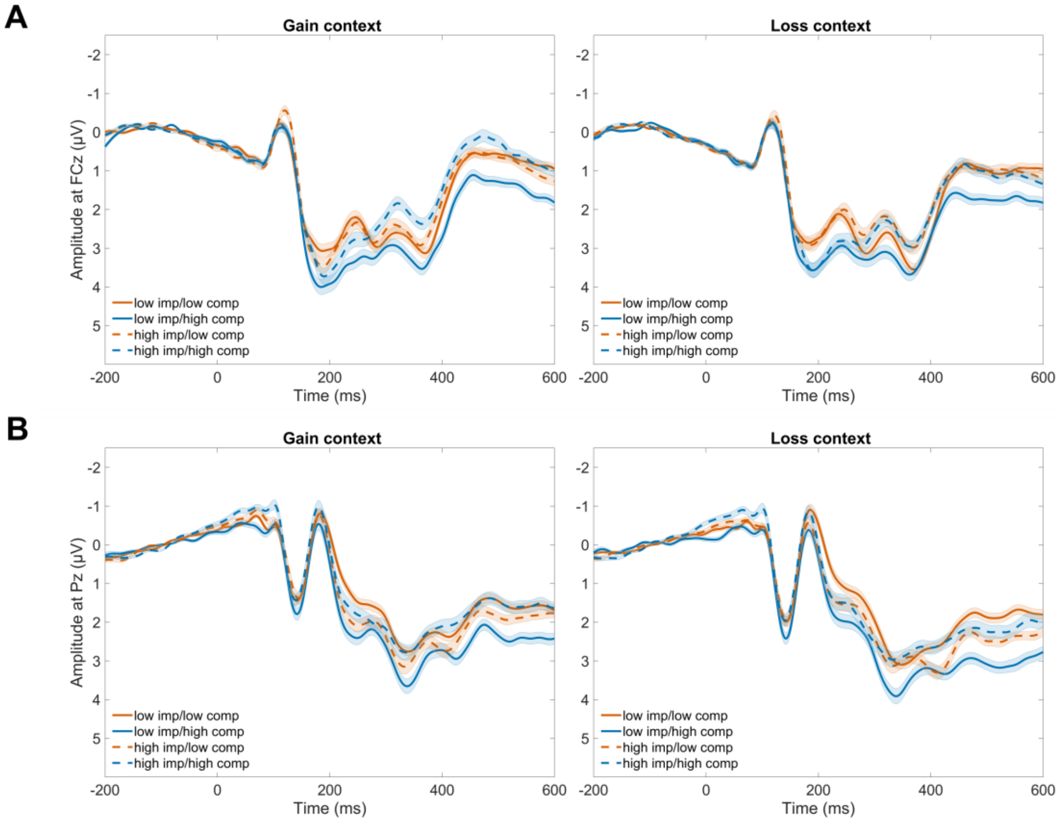

Fig. S5. *Time course of correct trials with POSITIVE FEEDBACK*. Impulsivity and compulsivity are split by median for visualization purposes into: low impulsive/low compulsive, low impulsive/high compulsive, high impulsive/low compulsive, and high impulsive/high compulsive. **A**, time course of feedback-locked correct trials with positive feedback at FCz, for the gain context and the loss context. **B**, time course of feedback-locked correct trials with positive feedback at Pz, for the gain context and the loss context. Shades indicate the SEM between subjects.

Discussion. Results suggest more attention is allocated to positive feedback in the gain context (P2 component) if impulsivity or compulsivity are high (Potts, 2004), higher motivational salience of gain (RewP) if compulsivity is high and impulsivity is low (Stewardson & Sambrook, 2023), and more attention in the loss context (P3a) if both impulsivity and compulsivity are low or high (Kirschner et al., 2022).

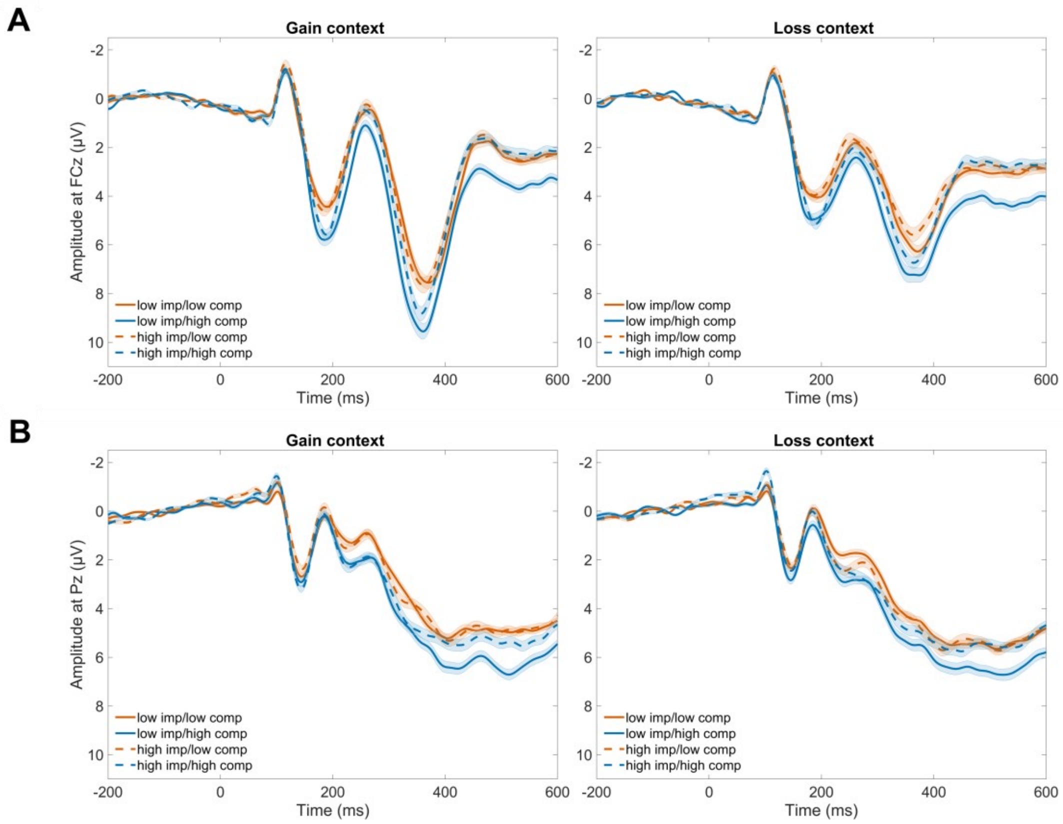

Fig. S6. *Time course of correct trials with NEGATIVE FEEDBACK*. Impulsivity and compulsivity are split by median for visualization purposes into: low impulsive/low compulsive, low impulsive/high compulsive, high impulsive/low compulsive, and high impulsive/high compulsive. **A**, time course of feedback-locked correct trials with negative feedback at FCz, for the potential gain context and the loss avoidance context. **B**, time course of feedback-locked correct trials with negative feedback at Pz, for the potential gain context and the loss avoidance context. Shades indicate the SEM between subjects.

References

Abramowitz, J. S., & Deacon, B. J. (2006). Psychometric properties and construct validity of the Obsessive–Compulsive Inventory—Revised: Replication and extension with a clinical sample. *Journal of anxiety disorders*, *20*(8), 1016-1035. https://doi.org/10.1016/j.janxdis.2006.03.001

Baguley, T. (2010). *interaction plot from cell means*. https://www.r-bloggers.com/2010/02/interaction-plot-from-cell-means/

Benjamini, Y., & Yekutieli, D. (2001). The control of the false discovery rate in multiple testing under dependency. *Annals of statistics*, *29*(4), 1165-1188. https://doi.org/10.1214/aos/1013699998

Cohen, J. D., Botvinick, M., & Carter, C. S. (2000). Anterior cingulate and prefrontal cortex: who's in control? *Nature neuroscience*, *3*(5), 421. https://doi.org/10.1038/74783

Fischer, A. G., Nigbur, R., Klein, T. A., Danielmeier, C., & Ullsperger, M. (2018). Cortical beta power reflects decision dynamics and uncovers multiple facets of post-error adaptation. *Nature communications*, *9*(1), 1-14. https://doi.org/10.1038/s41467-018-07456-8

Fischer, A. G., & Ullsperger, M. (2013). Real and fictive outcomes are processed differently but converge on a common adaptive mechanism. *Neuron*, *79*(6), 1243-1255. https://doi.org/10.1016/j.neuron.2013.07.006

Foa, E. B., Huppert, J. D., Leiberg, S., Langner, R., Kichic, R., Hajcak, G., & Salkovskis, P. M. (2002). The Obsessive-Compulsive Inventory: development and validation of a short version. *Psychological assessment*, *14*(4), 485. https://doi.org/10.1037/1040-3590.14.4.485

Gönner, S., Leonhart, R., & Ecker, W. (2007a). Das Zwangsinventar OCI-R-die deutsche Version des Obsessive-Compulsive Inventory-Revised. *PPmP-Psychotherapie· Psychosomatik· Medizinische Psychologie*, *57*(09/10), 395-404. https://doi.org/10.1055/s-2007-970894

Gönner, S., Leonhart, R., & Ecker, W. (2007b). The German version of the obsessive-compulsive inventory-revised: a brief self-report measure for the multidimensional assessment of obsessive-compulsive symptoms. *Psychotherapie, Psychosomatik, Medizinische Psychologie*, *57*(9-10), 395-404. https://doi.org/10.1055/s-2007-970894 (Das Zwangsinventar OCI-R - die deutsche Version des Obsessive-Compulsive Inventory-Revised - Ein kurzes Selbstbeurteilungsinstrument zur mehrdimensionalen Messung von Zwangssymptomen.)

Henry, J. D., & Crawford, J. R. (2005). The short-form version of the Depression Anxiety Stress Scales (DASS-21): construct validity and normative data in a large non-clinical sample. *British Journal of Clinical Psychology*, *44*(Pt 2), 227-239. https://doi.org/10.1348/014466505X29657

Kirschner, H., Fischer, A. G., & Ullsperger, M. (2022). Feedback-related EEG dynamics separately reflect decision parameters, biases, and future choices. *Neuroimage*, *259*, 119437. https://doi.org/10.1016/j.neuroimage.2022.119437

Klawohn, J., Meyer, A., Weinberg, A., & Hajcak, G. (2020). Methodological choices in event-related potential (ERP) research and their impact on internal consistency reliability and individual differences: An examination of the error-related negativity (ERN) and anxiety. *Journal of abnormal psychology*, *129*(1), 29. https://doi.org/10.1037/abn0000458

Long, J. A. (2022). Package 'interactions'.

Nilges, P., & Essau, C. (2015). Die Depressions-Angst-Stress-Skalen [The depression-anxiety-stress scales]. *Der Schmerz*, *29*(6), 649-657. https://doi.org/10.1007/s00482-015-0019-z

Overmeyer, R., & Endrass, T. (2023). Cognitive Symptoms Link Anxiety and Depression Within a Validation of the German State-Trait Inventory for Cognitive and Somatic Anxiety (STICSA). *Clinical Psychology in Europe*, *5*(2), e9753. https://doi.org/10.32872/cpe.9753

Patton, J. H., Stanford, M. S., & Barratt, E. S. (1995). Factor structure of the Barratt impulsiveness scale. *Journal of clinical psychology*, *51*(6), 768-774. https://doi.org/10.1002/1097-4679(199511)51:6<768::aid-jclp2270510607>3.0.co;2-1

Potts, G. F. (2004). An ERP index of task relevance evaluation of visual stimuli. *Brain and cognition*, *56*(1), 5-13. https://doi.org/10.1016/j.bandc.2004.03.006

R Core Team. (2022). *R: A language and environment for statistical computing*. In

Ree, M. J., French, D., MacLeod, C., & Locke, V. (2008). Distinguishing Cognitive and Somatic Dimensions of State and Trait Anxiety: Development and Validation of the State-Trait Inventory for Cognitive and Somatic Anxiety (STICSA). *Behavioural and Cognitive Psychotherapy*, *36*(3), 313-332. https://doi.org/10.1017/S1352465808004232

Santesso, D. L., & Segalowitz, S. J. (2009). The error-related negativity is related to risk taking and empathy in young men. *Psychophysiology*, *46*(1), 143-152. https://doi.org/10.1111/j.1469-8986.2008.00714.x

Schmidt, R. E., Gay, P., d'Acremont, M., & Van der Linden, M. (2008). A German Adaptation of the UPPS Impulsive Behavior Scale: Psychometric Properties and Factor Structure. *Swiss Journal of Psychology*, *67*(2), 107-112. https://doi.org/10.1024/1421-0185.67.2.107

Stanford, M. S., Mathias, C. W., Dougherty, D. M., Lake, S. L., Anderson, N. E., & Patton, J. H. (2009). Fifty years of the Barratt Impulsiveness Scale: An update and review. *Personality and Individual Differences*, *47*(5), 385-395. https://doi.org/10.1016/j.paid.2009.04.008

Stewardson, H., & Sambrook, T. D. (2023). Valence precedes value in neural encoding of prediction error. *Psychophysiology*, *60*(7), e14266. https://doi.org/10.1111/psyp.14266

The MathWorks Inc. (2018). *MATLAB*. In (Version version 9.5.0.944444 (R2018b))

Wickham, H., & Bryan, J. (2022). Read Excel Files [R package readxl version 1.4. 1]. *Published online August*, *17*.

Wickham, H., François, R., Henry, L., Müller, K., & Henry, L. (2022). RStudio. *Dplyr: A Grammar of Data Manipulation*.

Zambrano-Vazquez, L., & Allen, J. J. (2014). Differential contributions of worry, anxiety, and obsessive compulsive symptoms to ERN amplitudes in response monitoring and reinforcement learning tasks. *Neuropsychologia*, *61*, 197-209. https://doi.org/10.1016/j.neuropsychologia.2014.06.023

Zheng, Y., Sheng, W., Xu, J., & Zhang, Y. (2014). Sensation seeking and error processing. *Psychophysiology*, *51*(9), 824-833. https://doi.org/10.1111/psyp.12240
